# CircSATB1 Promotes Colorectal Cancer Liver Metastasis through Facilitating FKBP8 Degradation via RNF25‐Mediated Ubiquitination

**DOI:** 10.1002/advs.202406962

**Published:** 2025-02-08

**Authors:** Chuan Zhang, Chuanxin Tian, Renzhong Zhu, Chen Chen, Chi Jin, Xiaowei Wang, Lejia Sun, Wen Peng, Dongjian Ji, Yue Zhang, Yueming Sun

**Affiliations:** ^1^ Department of General Surgery The First Affiliated Hospital of Nanjing Medical University Colorectal Institute of Nanjing Medical University Nanjing 210000 China; ^2^ Institute of Translational Medicine, Medical College Yangzhou University Yangzhou 225000 China

**Keywords:** circSATB1, colorectal cancer, FKBP8, liver metastasis, RNF25

## Abstract

Colorectal cancer (CRC) is one of the most common cancers worldwide and liver metastasis is the leading reason for its mortality. Circular RNAs (circRNAs) are conclusively associated with the progression of various cancers, rendering the exploration of its specific mechanisms in colorectal cancer liver metastasis(CRLM) highly valuable. Combined with GEO (Gene Expression Omnibus) databases and clinical data in our center, we found that high expression of circSATB1 is closely related to the progression of CRLM. Functionally, circSATB1 could significantly promote the metastatic ability of CRC cells in vitro and in vivo. Mechanistically, circSATB1 facilitated the RNF25‐mediated ubiquitylation and degradation of FKBP8, releasing its inhibitory effects on mTOR signaling. In this process, circSATB1 acted as a scaffold for RNF25‐FKBP8 complexes. Additionally, circSATB1 could be packaged in exosomes and secreted from the CRC primary tumors into plasma. In conclusion, this study uncovered a new circSATB1 that acts as a potent promoter of CRLM and offers novel insights into the precision therapeutic strategies for CRLM.

## Graphical abstract

CircSATB1 is formed by the reverse splicing of exons 2–5 of the SATB1 linear transcript on chromosome 3. Mechanistically, circSATB1 facilitates the RNF25‐mediated ubiquitylation and degradation of FKBP8, releasing its inhibitory effects on mTOR signaling. In this process, circSATB1 acts as a scaffold for RNF25‐FKBP8 complexes. Additionally, circSATB1 can also be packaged in exosomes and secreted from the CRC cells into plasma to promote CRLM.

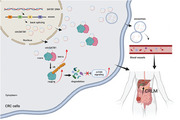

## 1 Introduction

Colorectal cancer (CRC) is one of the most common malignant tumors worldwide, posing a significant threat to human health due to its high morbidity and mortality.^[^ ^1^ ^]^

According to recent data, CRC has emerged as the second most frequently diagnosed cancer and the fourth leading cause of cancer‐related death in China.^[^ ^2^ ^]^ Notably, CRC frequently metastasizes to the liver, which is the most common site for distant metastasis of this disease.^[^ ^3^ ^]^ Unfortunately, patients with CRC liver metastasis (CRLM) have a considerably lower five‐year survival rate compared to those with localized CRC.^[^ ^4^, ^5^ ^]^

Despite the application of a comprehensive treatment regimen encompassing neoadjuvant radiotherapy, chemotherapy, targeted therapy, and immunotherapy, the prognosis for CRLM remains unsatisfactory.

Circular RNAs (circRNAs), circularized by a back‐splicing of pre‐mRNAs, are a class of conserved noncoding RNAs that possess a unique covalent closed‐loop structure without 5′ caps or 3′ tails. This distinctive structure renders circRNAs resistant to RNase R and thus more stable than linear RNAs.^[^ ^6^, ^7^ ^]^ Accumulating evidence suggests that circRNAs play a crucial role in regulating the differentiation, proliferation, and metastasis of tumor cells through various mechanisms.^[^ ^8^, ^9^, ^10^ ^]^ However, our understanding of the underlying mechanisms of circRNAs in CRLM remains at a nascent stage, necessitating further research and exploration.

SATB1 (Special AT‐rich sequence binding protein 1), a nuclear‐matrix‐binding protein containing 763 amino acids, was initially cloned because it can bind to the core unwinding element within the matrix attachment DNA region (MAR) at the 3′ ends of the immunoglobulin µ heavy chain (IgH) gene enhancer.^[^ ^11^ ^]^ As a genome organizer, SATB1 can specifically bind to AT‐rich DNA sequences, prompting DNA to fold into loops. Then, it recruits chromatin‐modifying enzymes to the DNA loops, thereby affecting gene transcription and playing a key role in the process of chromatin remodeling.^[^ ^12^ ^]^ SATB1 participates in various cellular biological processes such as proliferation, differentiation, apoptosis, and tumorigenesis through this mode of high‐order chromatin organization, underscoring its significance in cellular physiology.^[^ ^13^ ^]^ The abnormal expression of SATB1 has been confirmed to be closely related to the progression of various malignant tumors such as colorectal cancer, pancreatic cancer, liver cancer, kidney cancer, gastric cancer, bladder cancer, lung cancer, and so on.^[^ ^14^ ^]^ However, the role of circRNAs derived from SATB1 in CRC is not clear. Apart from the promotion of CRC progression by itself, it is worthy of exploration whether SATB1 can generate circRNAs through its splicing and further exert a role in CRC.

In this study, we have identified a novel circular RNA termed circSATB1. Notably, its expression was significantly upregulated in the primary tumors of patients with CRLM. Upon analyzing its correlation with clinicopathological factors, we observed a positive association between the expression level of circSATB1 and the TNM stage of CRC. Conversely, we found a negative correlation between circSATB1 expression and survival rates. Mechanistically, we discovered that circSATB1 enhanced the metastasis of CRC cells by promoting the ubiquitination and degradation of the FKBP8 protein. This process was catalyzed by the E3 ligase RNF25, leading to the activation of the mTOR signaling pathway. Additionally, circSATB1 could also be released via exosomes into the plasma, further contributing to the progression of CRLM. Thus, our findings reveal new perspectives on the underlying mechanisms of CRLM and identify novel potential therapeutic targets for the precise treatment.

## 2 Results

### 2.1 Upregulated circSATB1 Predicts Poor Prognosis and is Associated with the Progression of Liver Metastasis in CRC

To explore the role of circRNAs derived from SATB1 in the progression of CRLM, we consulted the circBank and circBase databases first and found that there are currently five known circRNAs derived from SATB1, including hsa_circ_0064554, hsa_circ_0064555, hsa_circ_0064556, hsa_circ_0064557, and hsa_circ_0064558.

**Figure 1:**
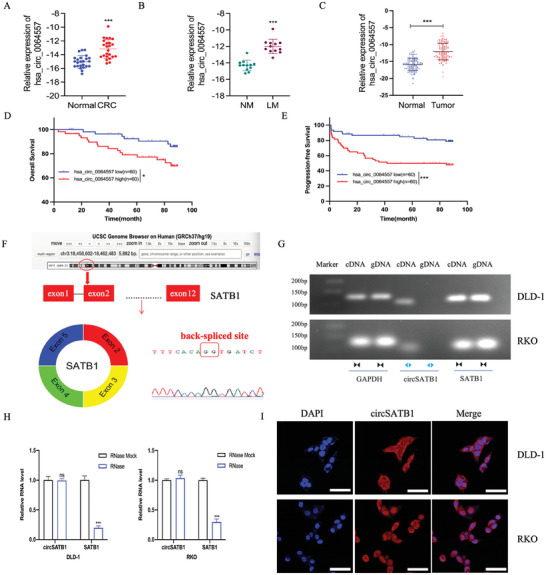
Aberrant expression of circSATB1 in CRC. A) The relative expression level(‐ΔCT) of hsa_circ_0064557 in adjacent normal tissues and CRC tissues from the 24 patients in cohort 1. n = 24 per group. B) The relative expression level(‐ΔCT) of hsa_circ_0064557 in CRC tissues from the 24 patients in the NM and LM group in cohort 1. n = 12 per group. C) hsa_circ_0064557 level detected by qRT‐PCR in 120 CRC tissues and matched adjacent tissues. n = 60 per group. D) The OS of 120 CRC patients with different circSATB1 levels is shown by Kaplan–Meier curves. n = 60 per group. E) The PFS of 120 CRC patients with different circSATB1 levels is shown by Kaplan–Meier curves. n = 60 per group. F) Schematic illustration and Sanger sequencing of circSATB1. G) cDNA and gDNA of CRC cells were amplified with convergent primers for linear SATB1 RNA and with divergent primers for circSATB1. GAPDH was the negative control. H) The qRT‐PCR analysis for circSATB1 and linear SATB1 mRNA in CRC cells with or without RNase R treatment. I) The subcellular localization of circSATB1(red) in CRC cells indicated by RNA‐FISH, with nuclei stained with DAPI (blue) (Scale bar, 50 µm). Data are presented as mean ± SD in at least three independent experiments. ^*^ *P* < 0.05, ^**^ *P* < 0.01, ^***^ *P* < 0.001, and *P* > 0.05, not significant (n.s.). Student's t‐test (A, B, C, and H) and log‐rank test (D, E) were used to determine statistical significance.

The GSE147597 dataset in the Gene Expression Omnibus (GEO), which detects the expression profiles of circRNAs in 10 pairs of CRC tissues with or without liver metastasis (LM), was performed differential expression analysis (|log FC|>1, adj. *P* < 0.05). The results showed that there are two circRNAs derived from SATB1 in the dataset: hsa_circ_0064555 and hsa_circ_0064557. Among them, hsa_circ_0064557 was upregulated in the LM group compared to the NM(non‐metastasis) group (log FC = 1.2997, adj. *P* < 0.01), while there was no difference in the expression level of hsa_circ_0064555 between the two groups (log FC = −0.3582, adj. *P*>0.05) (Figure S1A,B, Supporting Information). We then detected the expression levels of the five circRNAs derived from SATB1 in CRC and paired normal tissues of randomized 24 patients (12 NM and 12 LM patients) in cohort 1 by qRT‐PCR. We found that the expression level of hsa_circ_0064557 in CRC tissues was significantly higher than the paired normal tissues, and was significantly higher in CRC tissues in the LM group than NM group (**Figure** **1**A,B; Figure S1C–J, Supporting Information).

In cohort 2, hsa_circ_0064557 was detected in CRC and paired normal tissues from randomized 120 CRC patients with or without liver metastasis by qRT‐PCR. The results showed that hsa_circ_0064557 was significantly overexpressed in the CRC tissues than the paired normal tissues (Figure 1C). These 120 patients were divided into two groups by the median level of hsa_circ_0064557 (low expression group and high expression group). We discovered a positive correlation between hsa_circ_0064557 and various factors, including TNM stage, liver metastasis, vascular and nerve invasion, as well as CEA levels (Table S1, Supporting Information). Furthermore, the Overall Survival (OS) and Progression‐free Survival (PFS) rates of patients in the high‐expression group were inferior to those in the other group (Figure 1D,E). These results indicated that hsa_circ_0064557 was significantly correlated with the progression of CRLM and unfavorable prognosis. The sequence alignment results suggested that hsa_circ_0064557 was formed by the reverse splicing of exons 2 to 5 of SATB1 linear transcript on chromosome 3, containing 663 nucleotides (termed as circSATB1). Sanger sequencing confirmed the head‐to‐tail splicing point of circSATB1 via the PCR products amplified by divergent primers (Figure 1F). Agarose electrophoresis experiments showed that circSATB1 could only amplified by divergent primers from cDNA but not gDNA in CRC cells (Figure 1G). Moreover, circSATB1 but not linear SATB1 was resistant to RNase R treatment (Figure 1H).

FISH (Fluorescence in situ hybridization) assays confirmed that circSATB1 was localized mainly in the cytoplasm of CRC cells (Figure 1I). These results indicated that circSATB1 was a stable circular RNA in CRC cells.

### 2.2 CircSATB1 Promotes the Metastasis of CRC Cells In Vitro and Vivo

**Figure 2:**
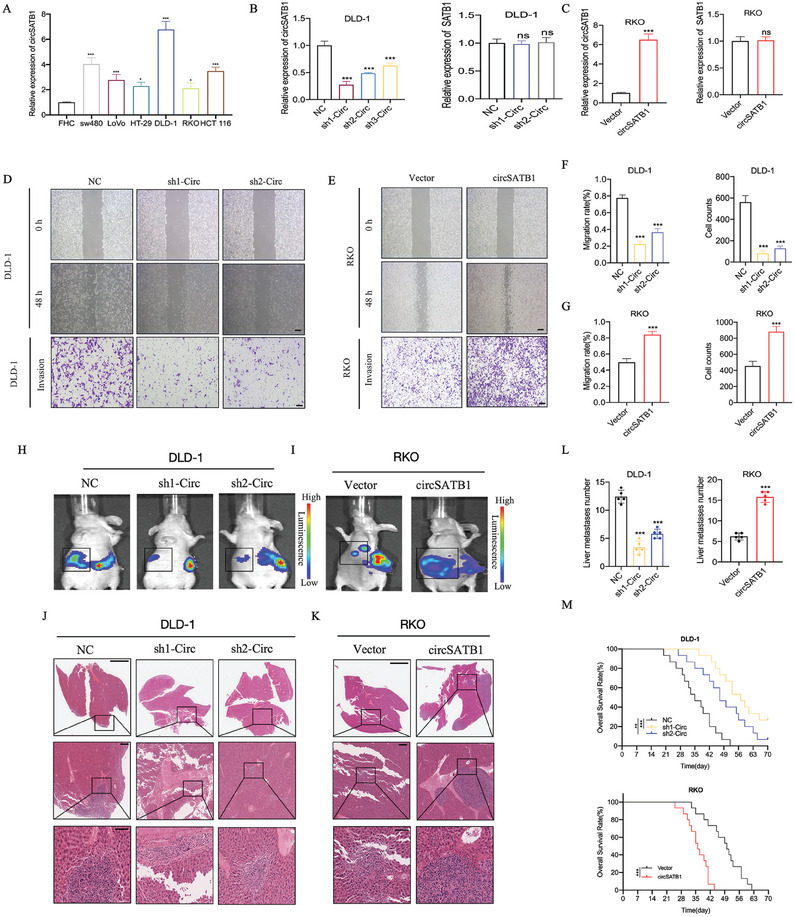
CircSATB1 promotes the metastasis of CRC cells in vitro and vivo. A) CircSATB1 relative expression level(2^−ΔΔCT^) detected by qRT‐PCR in FHC and CRC cells. B) Relative expression of CircSATB1 and SATB1 mRNA(2^−ΔΔCT^) detected by qRT‐PCR in DLD‐1 cells transfected with NC or sh‐Circ. C) Relative expression of CircSATB1 and SATB1 mRNA(2^−ΔΔCT^) detected by qRT‐PCR in RKO cells transfected with Vector or circSATB1. D–G) The effects of circSATB1 on migratory and invasive capabilities of DLD‐1 and RKO cells were detected by wound healing and transwell assays in vitro(Wound Healing, Scale bar, 200 µm; Transwell, Scale bar, 100 µm). H, I) Representative bioluminescence images of mice (n = 5 per group) injected with different transfected DLD‐1 and RKO cells. J, K) Representative HE staining images of the liver metastasis lesions. n = 5 per group (Top, Scale bar, 4 mm; Middle, Scale bar, 600 µm; Bottom, Scale bar, 200 µm). L) The number of the liver metastatic foci. n = 5 per group. M) The OS of mice injected with different transfected DLD‐1 and RKO cells, showed by Kaplan–Meier curves. n = 15 per group. Data are presented as mean ± SD in at least three independent experiments. *P* < 0.05 is considered statistically significant. ^*^ *P* < 0.05, ^**^ *P* < 0.01, and ^***^ *P* < 0.001, *P* > 0.05, not significant (n.s.). One‐way ANOVA test (A, B, F, L left), Student's t‐test (C, G, L right), and log‐rank test (M) were used to determine statistical significance.

To explore the functions of circSATB1 in CRC cells, we first detected the relative expression of circSATB1 in FHC and CRC cells and found that circSATB1 was up‐regulated in CRC cell lines (**Figure** **2**A). According to this data, DLD‐1 and RKO cells were selected for further investigation. The short hairpin (sh)RNAs (NC, sh1‐Circ, sh2‐Circ, and sh3‐Circ) or plasmids(Vector, circSATB1) were stably transfected into DLD‐1 or RKO cells through the lentivirus infection system, respectively. The qRT‐PCR findings validated the transfection efficiency and unequivocally confirmed that the expression level of linear SATB1 remained stable, unaffected by circSATB1 (Figure 2B,C). In vitro, wound healing and transwell assays confirmed that silencing circSATB1 dramatically weakened the migration and invasion of CRC cells, while circSATB1 overexpression strengthened these abilities (Figure 2D–G). In vivo, bioluminescent signals showed that overexpression of circSATB1 promoted the liver metastasis of RKO cells, whereas knockdown of circSATB1 drastically diminished the liver metastasis capabilities of DLD‐1 cells (Figure 2H,I and S4A,B, Supporting Information). This observation was further corroborated by hematoxylin and eosin (HE) staining and number statistics (Figure 2J–L). Notably, the knockdown of circSATB1 significantly prolonged the OS of the mice, whereas its overexpression markedly shortened their OS (Figure 2M). Given that the activation of epithelial‐mesenchymal transition (EMT) is associated with the metastasis of tumors, we confirmed the oncogenic role of circSATB1 by detecting the expression level of EMT‐related proteins. The results indicated that silencing circSATB1 resulted in a decrease in the expression level of vimentin protein and an increase in the expression level of E‐cadherin protein. Upon overexpressing circSATB1, the expression levels of the two proteins changed in opposite directions, specifically (Figure S4G, Supporting Information). Overall, these results showed that circSATB1 promoted the metastasis of CRC cells in vitro and in vivo.

### 2.3 CircSATB1 Interacts with FKBP8 to Reduce its Protein Level in a Post‐Transcriptional Manner

**Figure 3:**
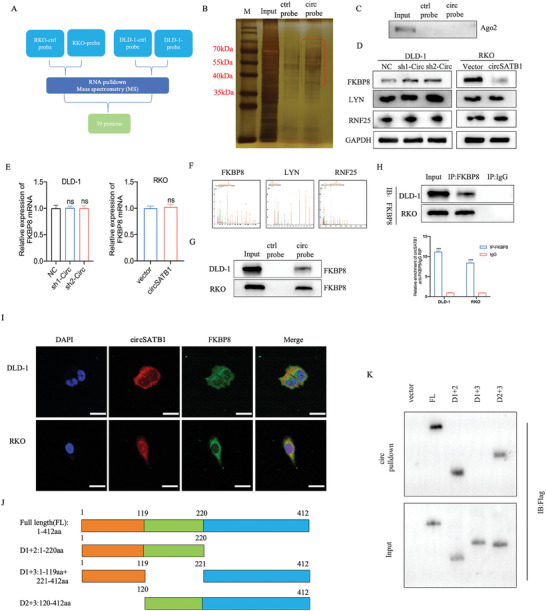
CircSATB1 physically interacts with FKBP8 protein and reduces its level in a post‐transcriptional manner. A) The potential circSATB1‐interacting proteins detected by RNA pull‐down assay and MS. B) The specific proteins interacting with circSATB1 detected by SDS‐PAGE and silver staining. C) Ago2 was detected by WB with the precipitates generated from the pull‐down assay. D) The effects of circSATB1 on the protein level of FKBP8, LYN, and RNF25 were detected by WB. E) The effects of circSATB1 on the mRNA level of FKBP8 detected by qRT‐PCR. F) The representative peptide fragments of FKBP8, LYN, and RNF25 identified by MS. G, H) The RNA pull‐down products detected by WB with anti‐FKBP8 (G) and immunoprecipitated circSATB1 with anti‐FKBP8 analyzed by RIP‐PCR assay (H). I) The colocalization of circSATB1 (red) and FKBP8 (green) in CRC cells indicated by RNA‐FISH and immunofluorescence staining assay, with nuclei stained with DAPI (blue) (Scale bar, 50 µm). J) Schematic illustration of FKBP8 truncations. K) Immunoblotting analysis showed the essential region of FKBP8 for the interaction with circSATB1. Data are presented as mean ± SD in at least three independent experiments. *P* < 0.05 is considered statistically significant (^*^ *P* < 0.05, ^**^ *P* < 0.01, and ^***^ *P* < 0.001); *P* > 0.05, is not significant (n.s.). One‐way ANOVA test (E left) and Student's t‐test (E right and H) were used to determine statistical significance.

To further explore the mechanisms of circSATB1 in CRC cells, we performed RNA pull‐down in DLD‐1 and RKO cells using a biotin‐labeled specific probe of circSATB1.The precipitates generated from the pull‐down assay were analyzed by mass spectrometry (MS), revealing that the circSATB1 probe was able to specifically pull down a total of 1807 proteins in DLD‐1 cells and 3258 proteins in RKO cells, respectively. Among the identified proteins, there were 360 and 744 specific proteins detected in DLD‐1 and RKO cells, respectively, excluding those proteins pulled down by the negative control probe. Venn diagram showed that 39 circSATB1‐interacting proteins overlapped in RKO and DLD‐1 cells (**Figure** **3**A). SDS‐PAGE and silver staining were also used to detect the specific proteins interacting with circSATB1 (Figure 3B). Ago2, a crucial effector of the ceRNA mechanism, was neither pulled down by the circSATB1 probe nor detected through Western blotting (WB) (Figure 3C). This data revealed that circSATB1 did not play the role of miRNA sponge in CRC cells. According to the silver staining results, there were specific protein bands between 35 and 70 kDa. Within this range, there were 12 proteins (Table S2, Supporting Information). When ranked according to the abundance, three specific proteins (RNF25, LYN, FKBP8) were found to be among the top 6 in both cell lines simultaneously. Numbers of researches have indicated that circRNAs could regulate the stability of their binding proteins.^[^ ^15^, ^16^ ^]^ To verify whether circSATB1 could affect the stability of RNF25, LYN, and FKBP8 protein, we detected their mRNA and protein levels in CRC cells with circSATB1 knockdown or overexpression. The results showed that overexpression of circSATB1 decreased the level of FKBP8 protein but had no significant influence on its mRNA level. Conversely, the knockdown of circSATB1 led to an increase in FKBP8 protein level, yet did not alter its mRNA level. Besides, circSATB1 had no effects on either the protein or mRNA level of RNF25 or LYN (Figure 3D,E and S1K–N, Supporting Information).WB and RIP‐PCR assays confirmed the interaction between circSATB1 and FKBP8 protein (Figure 3F–H). Additionally, FISH and immunofluorescence (IF) assays suggested that circSATB1 was co‐localized with FKBP8 protein in the cytoplasm of CRC cells (Figure 3I). Domain mapping analysis demonstrated that Domain 2(120‐220aa) of FKBP8 was the essential region for the interaction with circSATB1 (Figure 3J,K). Taken together, circSATB1 could physically interact with FKBP8 protein and reduce its level in a post‐transcriptional manner in CRC cells.

### 2.4 CircSATB1 Exerts an Oncogenic Role Mediated by FKBP8 in CRC Cells

IHC (Immunohistochemistry) was performed to analyze the protein level of FKBP8 with the clinical characters of the 56 CRC patients with or without liver metastasis in cohort 3. The results showed that FKBP8 was negatively correlated to the TNM stage of CRC (Table S3, Supporting Information). To investigate whether circSATB1 impacts CRC cells via regulating FKBP8, we performed rescue experiments. First, we performed qRT‐PCR to verify the knockdown or overexpression efficiency of FKBP8 and confirmed that sh2‐FKBP8 and sh3‐FKBP8 could significantly reduce the expression level of FKBP8 mRNA while oe‐FKBP8 had the opposite effects. However, the expression level of circSATB1 and linear SATB1 mRNA did not change significantly (Figure S2A–C, Supporting Information). At the protein level, WB results confirmed that sh2‐FKBP8 and sh3‐FKBP8 could significantly reduce the level of FKBP8 protein while oe‐FKBP8 played the opposite role. Additionally, sh2‐FKBP8 could weaken the effects of knockdown of circSATB1 on the increase of FKBP8 protein level while oe‐FKBP8 could partially restore the effects of overexpression of circSATB1 on the reduction of FKBP8 protein level (Figure S2D,E, Supporting Information).

**Figure 4:**
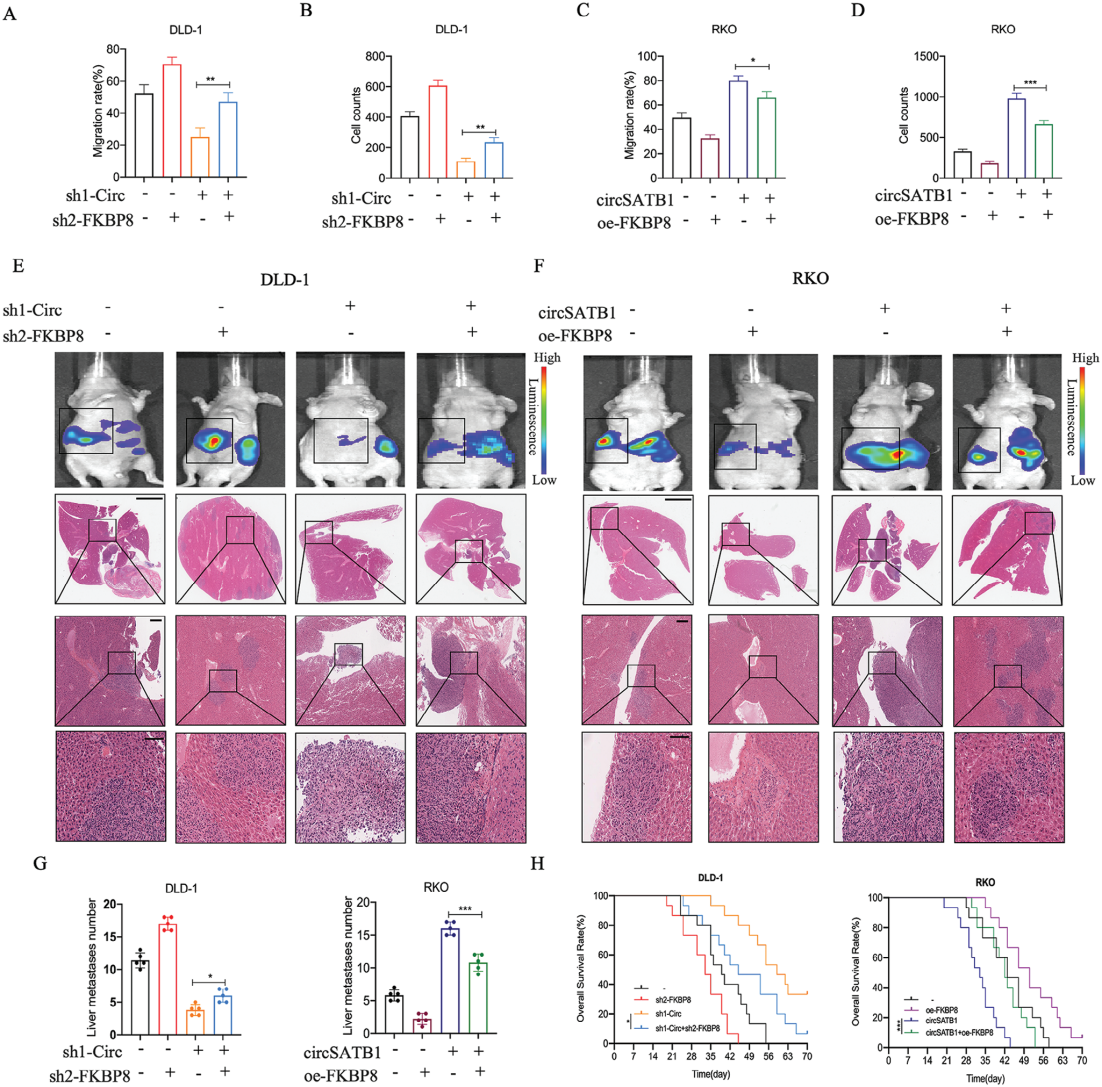
CircSATB1 exerts an oncogenic role mediated by FKBP8 in CRC cells. A–D) The reversal effects of FKBP8 on the migration and invasion capacity of DLD‐1 and RKO cells are shown by wound healing and transwell assays in vitro. E, F) Representative bioluminescence images of mice (n = 5 per group) injected with different transfected DLD‐1 and RKO cells and representative HE staining images of the liver metastasis lesions (n = 5 per group) (Top, Scale bar, 4 mm; Middle, Scale bar, 600 µm; Bottom, Scale bar, 200 µm). G) The number of the liver metastatic foci. n = 5 per group. H) The OS of mice injected with different transfected DLD‐1 and RKO cells, showed by Kaplan–Meier curves. n = 15 per group. Data are presented as mean ± SD in at least three independent experiments. *P* < 0.05 is considered statistically significant. ^*^ *P* < 0.05, ^**^ *P* < 0.01, and ^***^ *P* < 0.001, *P* > 0.05, not significant (n.s.). One‐way ANOVA tests (A, B, C, D, and G) and log‐rank tests (H) were used to determine statistical significance.

In vitro, FKBP8 knockdown could mitigate the inhibitory effects on cell migration and invasion in DLD‐1 cells that were originally induced by the knockdown of circSATB1. Additionally, overexpressing FKBP8 counteracted the enhancing effects on the metastasis capacity in RKO cells, which were caused by the overexpression of circSATB1 (**Figure** **4**A–D; Figure S2F,G, Supporting Information). The same trend was also observed in liver metastasis models in vivo, which was confirmed by bioluminescent signals and HE staining (Figure 4E–G; Figure S4C,D, Supporting Information). Similarly, the OS of the mice also showed that FKBP8 knockdown or overexpression could reverse the effects induced by circSATB1(Figure 4H). These findings indicated that circSATB1 could exert an oncogenic role mediated by FKBP8 in CRC cells.

### 2.5 CircSATB1 Promotes the Proteasomal Degradation of FKBP8 Protein

**Figure 5:**
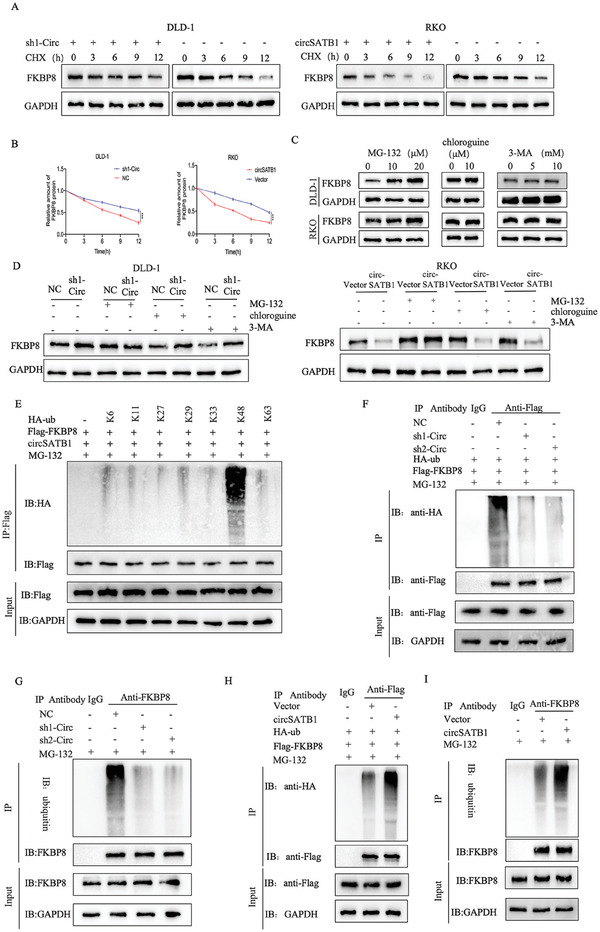
CircSATB1 promotes the proteasomal degradation of FKBP8. A, B) The expression levels of FKBP8 protein detected by IB in different transfected DLD‐1 or RKO cells. The cells were treated with cycloheximide (CHX, 100 µg mL^−1^) for the indicated time points before harvesting. C) The level of FKBP8 protein detected via WB in DLD‐1 or RKO cells treated with different doses of MG‐132, chloroquine, or 3‐MA for 8 h before harvesting. D) The effects of MG‐132(20µm, 8 h), chloroquine(10µm, 8 h) or 3‐MA(10mm, 8 h) on FKBP8 protein stability in different transfected DLD‐1 or RKO cells. E) IB analysis of IP products with anti‐Flag derived from lysates of HEK‐293T cells transfected with different constructs. F) IP analysis of lysates from HEK‐293T cells transfected with HA‐Ub, Flag‐FKBP8 with NC or sh1‐Circ or sh2‐Circ. G) IB analysis of lysates from DLD‐1 cells transfected with NC or sh1‐Circ or sh2‐Circ. H) IB analysis of lysates from HEK‐293T cells transfected with HA‐Ub, Flag‐FKBP8 with Vector or circSATB1. I) IB analysis of lysates from RKO cells transfected with Vector or circSATB1. All the cells(E–I) were treated with 20 µm MG‐132 for 8 h before harvesting. Data are presented as mean±SD in three independent experiments. *P* < 0.05 is considered statistically significant. ^***^ *P* < 0.001. Student's t‐test (B) was used to determine statistical significance.

Given that circSATB1 modified FKBP8 protein in a post‐transcriptional manner, we treated CRC cells with Cycloheximide (CHX, inhibitor of protein synthesis) and analyzed the half‐life of FKBP8. The results showed that overexpression of circSATB1 significantly reduced the half‐life of FKBP8 in RKO cells. In contrast, the application of sh1‐Circ in DLD‐1 cells led to a significant prolongation of the half‐life of FKBP8 (**Figure** **5**A,B).Ubiquitin–proteasome system(UPS) and autophagy‐lysosome system are two important systems to adjust protein degradation in the post‐transcriptional manner.^[^ ^17^, ^18^ ^]^ Thus, we treated CRC cells with proteasome inhibitor MG‐132, the lysosome inhibitor chloroquine, or the autophagy inhibitor 3‐Methyladenine (3‐MA) to determine which was the pivotal system. Instead of chloroquine or 3‐MA, MG‐132 treatment could slow down the degradation of FKBP8 protein in a dose‐dependent manner (Figure 5C). Additionally, WB showed that MG‐132 treatment, but not chloroquine or 3‐MA, moderated the difference in FKBP8 protein degradation between NC and sh1‐Circ DLD‐1 cells. Analogously, only MG‐132 could dramatically reverse the degradation of FKBP8 protein whether circSATB1 overexpression or not in RKO cells (Figure 5D). These results suggested that circSATB1 mainly regulated the stability of FKBP8 protein through UPS.

To detect which type of ub chains were involved in the ubiquitination of FKBP8, we designed constructs with only one wide lysine(K) and others mutated to arginine(R). The IB (Immunoblotting) results showed that FKBP8 was significantly modified by the K48‐linked ubiquitin chain (Figure 5E). Furthermore, the ubiquitination assays showed that silencing circSATB1 could reduce the endogenous ubiquitination level of FKBP8 in DLD‐1cells and circSATB1 overexpression played the opposite effects in RKO cells. The exogenous data in HEK‐293T cells showed the same results (Figure 5F–I). These findings demonstrated that circSATB1 promoted the ubiquitination/proteasomal degradation of FKBP8.

### 2.6 RNF25 Mediates FKBP8 Ubiquitination as an E3 Ubiquitin Ligase

**Figure 6:**
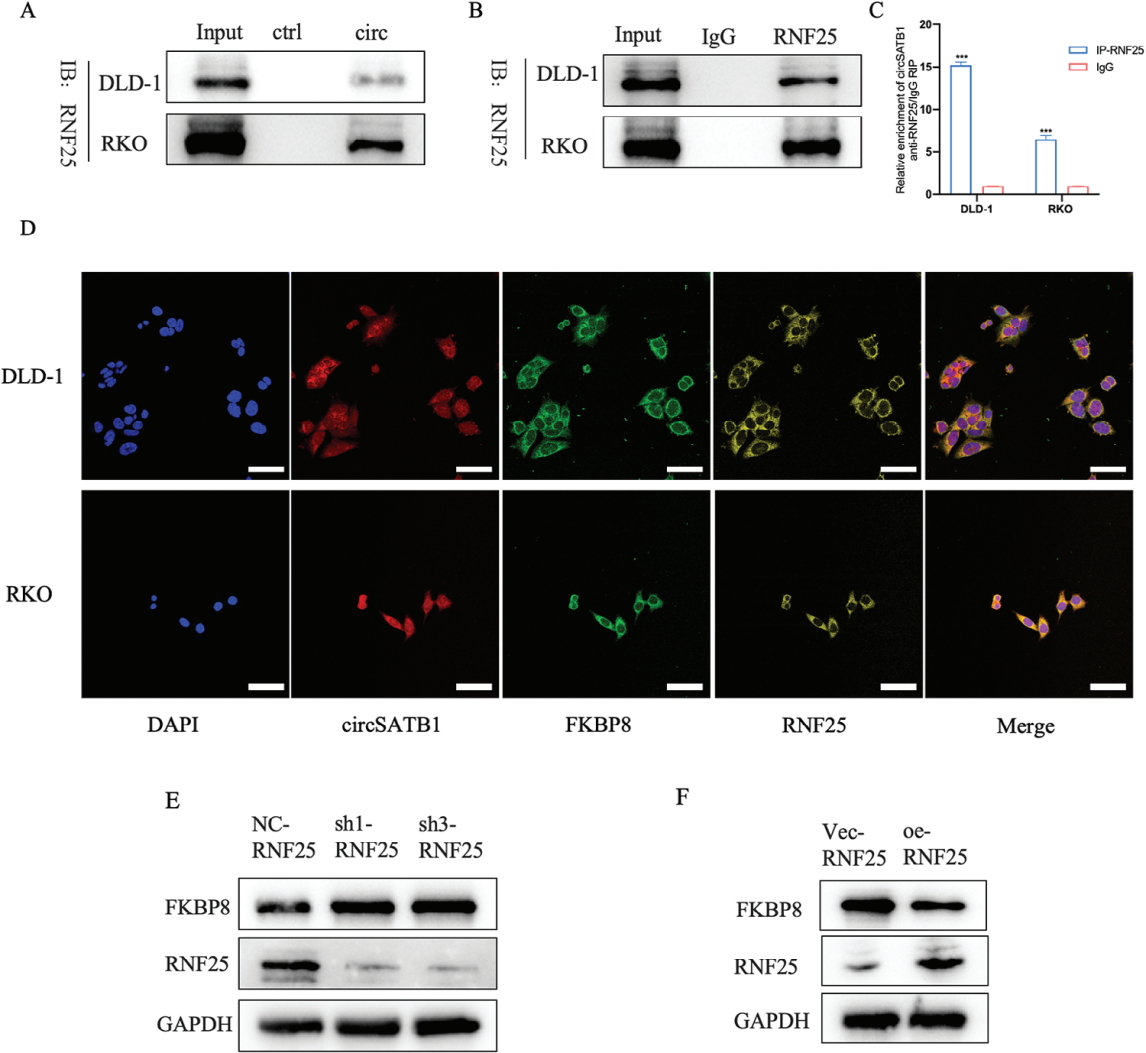
RNF25 physically interacts with FKBP8 protein and regulates its level negatively. A) The RNA pull‐down products detected by IB with anti‐RNF25. B, C) The IP products with anti‐RNF25 were detected by IB and the relative enrichment of circSATB1 was detected by qRT‐PCR(2^−ΔΔCT^). D) The colocalization of circSATB1 (red), FKBP8 (green), and RNF25 (yellow) in CRC cells indicated by FISH and IF staining assay, with nuclei stained with DAPI (blue) (Scale bar, 50 µm). E) The protein level of FKBP8 and RNF25 is regulated by RNF25 knockdown. F) The protein level of FKBP8 and RNF25 is regulated by RNF25 overexpression. Data are presented as mean ± SD in three independent experiments. *P* < 0.05 is considered statistically significant. ^***^ *P* < 0.001. Student's t‐test (C) was used to determine statistical significance.

RNA pull‐down and MS analysis showed that there were two E3 ligases, RNF25 and NEDD4L, among the 39 potential interacting proteins. Combined with the previous analysis, RNF25 might participate in the turnover of FKBP8 through UPS. In this study, RNA pulldown, IB and RIP assays confirmed that RNF25 could specifically bind to circSATB1 (**Figure** **6**A–C).IF staining and FISH assays showed that circSATB1, RNF25, and FKBP8 mainly co‐localized in the cytoplasm of CRC cells (Figure 6D).

Comprehensive analysis of IHC results and clinical information of CRC patients in cohort 3 showed that RNF25 was positively associated with advanced TNM stage (Table S4, Supporting Information). To detect the function of RNF25 between FKBP8 and circSATB1, we constructed stable DLD‐1 and RKO cell lines with RNF25 knockdown or overexpression. The verification of the interference efficiency is shown in Figure S3A,B (Supporting Information). We found that RNF25 knockdown could increase FKBP8 protein levels and RNF25 overexpression played the opposite effects (Figure 6E,F). However, FKBP8 mRNA levels accompanied by circSATB1 and SATB1 linear RNA levels could not be affected by RNF25 (Figure S3C,D, Supporting Information). Additionally, WB showed that the change of FKBP8 had no significant effects on the expression of RNF25 protein (Figure S3E,F, Supporting Information).

**Figure 7:**
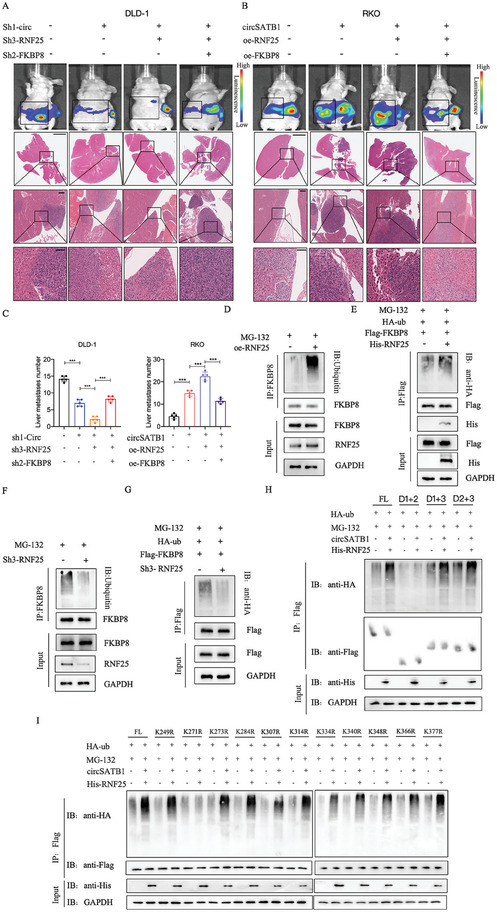
RNF25 mediates FKBP8 ubiquitination as an E3 ubiquitin ligase. A, B) Representative bioluminescence images of mice (n = 5 per group) injected with different transfected DLD‐1 and RKO cells and representative HE staining images of the liver metastasis lesions(n = 5 per group). The data showed the co‐effects of circSATB1, RNF25, and FKBP8 on the liver metastasis ability of DLD‐1 and RKO cells in vivo(Top, Scale bar, 4 mm; Middle, Scale bar, 600 µm; Bottom, Scale bar, 200 µm). C) The number of the liver metastatic foci.n = 5 per group. D) IP‐FKBP8 and IB analysis of lysates from DLD‐1 cells transfected with oe‐RNF25 or not. E) IP‐Flag and IB analysis of lysates from HEK‐293T cells transfected with HA‐Ub, Flag‐FKBP8 with His‐RNF25 or not. F) IP‐FKBP8 and IB analysis of lysates from RKO cells transfected with sh3‐RNF25 or not. G) IP‐Flag and IB analysis of lysates from HEK‐293T cells transfected with HA‐Ub, Flag‐FKBP8 with sh3‐RNF25 or not. H) IP‐Flag and IB analysis indicated the ubiquitination of the Flag‐FKBP8 truncated constructs in HEK‐293T cells co‐transfected with HA‐ub, circSATB1and His‐RNF25 or not. I) IP‐Flag and IB analysis of lysates from HEK‐293T cells transfected Flag‐FKBP8 full‐length construct or indicated mutant constructs, together with HA‐ub, circSATB1, His‐RNF25 or not. All the cells (D–I) were treated with 20 µm MG‐132 for 8 h before harvesting. Data are presented as mean ± SD in three independent experiments. *P* < 0.05 is considered statistically significant. ^***^ *P* < 0.001. One‐way ANOVA test (C) was used to determine statistical significance.

The phenotypic analysis of CRC cells, both in vitro and in vivo, confirmed the following observations:knockdown of RNF25 further diminished the metastatic capabilities of DLD‐1 cells, already weakened by the silencing of circSATB1, yet this effect was mitigated by knocking down FKBP8. Conversely, the overexpression of RNF25 augmented the metastatic ability of RKO cells, which was already enhanced by the overexpression of circSATB1. However, this enhancement was attenuated by the overexpression of FKBP8 (**Figure** **7**A–C; Figure S3G–L, Figure S4E,F, Supporting Information). Furthermore, the ubiquitination level of FKBP8 could be positively regulated by RNF25 (Figure 7D–G). The ubiquitination assays with truncated constructs showed that Domain 3(221‐412aa) of FKBP8 was the main region modulated by RNF25 (Figure 7H). To further detect the specific ubiquitination sites, we analyzed the Metosite, Phospho‐SitePlus, and iPTMnet databases and predicted 11 potential ubiquitination sites within Domain 3 of the FKBP8 protein. We then constructed the FKBP8 mutants with lysine (K)‐to‐arginine (R) substitution for every potential lysine residue. The ubiquitination assays indicated that K271 was the unique residue ubiquitinated by RNF25 (Figure 7I). These results suggested that RNF25 could interact with FKBP8 protein as the E3 ubiquitin ligase to promote its ubiquitination and degradation.

### 2.7 CircSATB1 Acts as a Modular Scaffold for RNF25 and FKBP8 Protein Complex

**Figure 8:**
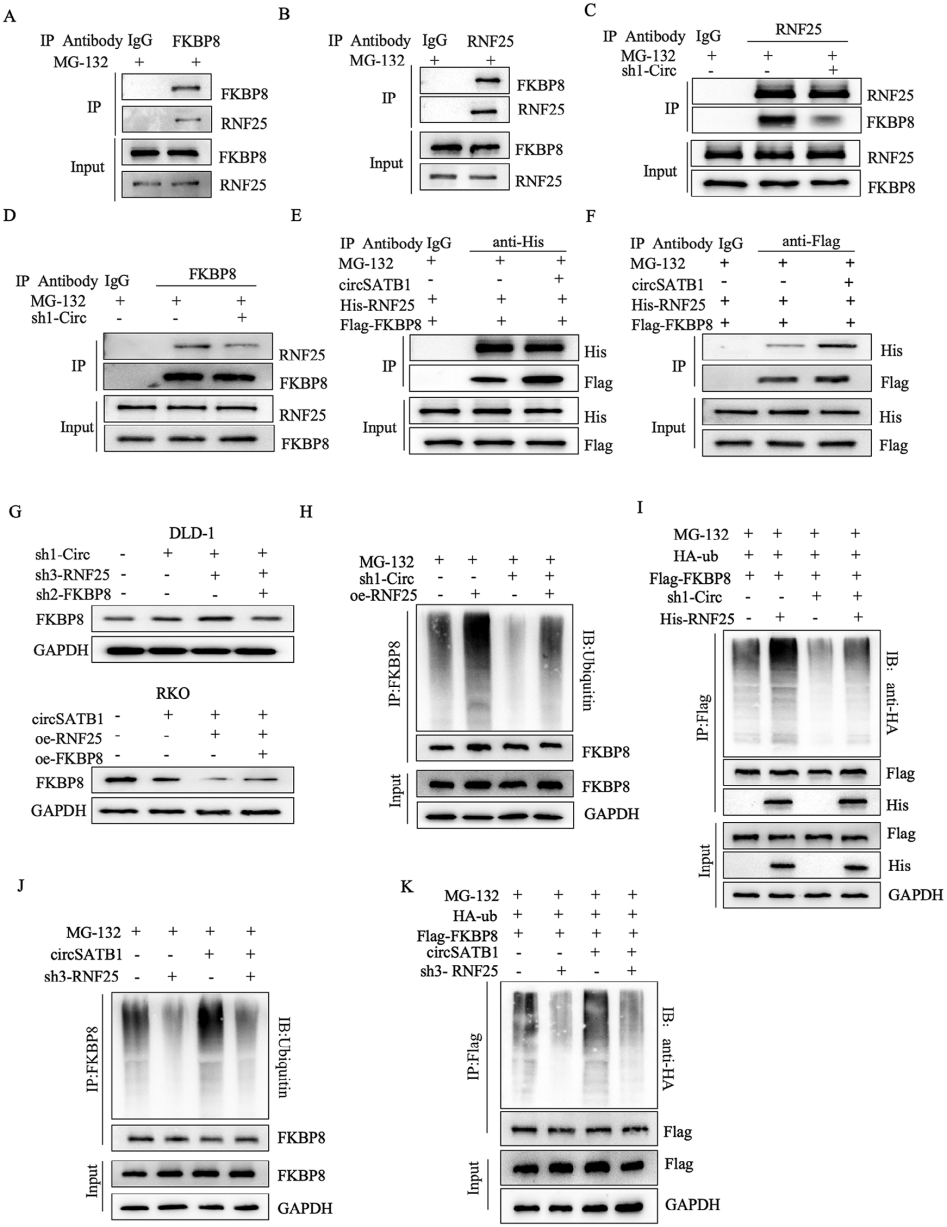
CircSATB1 acts as a modular scaffold for RNF25 and FKBP8 protein complex. A, B) Co‐IP and IB assays showing the interaction of FKBP8 and RNF25 in DLD‐1 cells. C) IP‐RNF25 and IB assays showing the effects of sh1‐Circ on the abundance of FKBP8 protein interacted with RNF25 in DLD‐1 cells. D) IP‐FKBP8 and IB assays showing the effects of sh1‐Circ on the abundance of RNF25 protein interacted with FKBP8 in DLD‐1 cells. E) IP‐His and IB assays showing the effects of circSATB1 on the abundance of FKBP8 protein interacted with RNF25 in HEK‐293T cells transfected with His‐RNF25, Flag‐FKBP8 with circSATB1 or not. F) IP‐Flag and IB assays showing the effects of circSATB1 on the abundance of RNF25 protein interacted with FKBP8 in HEK‐293T cells transfected with His‐RNF25, Flag‐FKBP8 with circSATB1 or not. G) The effects of circSATB1, RNF25, and FKBP8 working together on FKBP8 protein. H) The ubiquitination level of FKBP8 was detected by IP‐FKBP8 and IB analysis of lysates from DLD‐1 cells transfected with sh1‐Circ, or‐RNF25 or not. I) IP‐Flag and IB analysis of lysates from HEK‐293T cells transfected with HA‐Ub, Flag‐FKBP8, sh1‐Circ, His‐RNF25 or not. J) The ubiquitination level of FKBP8 protein detected by IP‐FKBP8 and IB analysis of lysates from RKO cells transfected with circSATB1, sh3‐RNF25 or not. K) IP‐Flag and IB analysis of lysates from HEK‐293T cells transfected with HA‐Ub, Flag‐FKBP8, circSATB1, sh3‐RNF25 or not. All the cells (A, B, C, D, E, F, H, I, J, K) were treated with 20 µm MG‐132 for 8 h before harvesting.

Given that circSATB1 could interact with RNF25 and FKBP8, we speculated that circSATB1 may play as a modular scaffold for the protein complex. First, the co‐IP assays showed that RNF25 could interact with FKBP8 protein directly (**Figure** **8**A,B).

To confirm the assumption of the modular scaffold, we performed co‐IP assays in DLD‐1 and HEK293T cells with circSATB1 knockdown or overexpression. The results showed that circSATB1 knockdown reduced the binding abundance between FKBP8 and RNF25 proteins (Figure 8C,D) while circSATB1 overexpression played the opposite role (Figure 8E,F).WB showed that RNF25 could regulate the expression of FKBP8 protein in the same trend with circSATB1 (Figure 8G). Silencing circSATB1 weakened the ubiquitination level of FKBP8 protein mediated by RNF25, however, circSATB1 overexpression had the opposite effects (Figure 8H–K). These findings indicated that circSATB1 acts as an intermediary to enhance the binding ability between FKBP8 and RNF25 and then promotes RNF25‐mediated FKBP8 ubiquitination/proteasomal degradation.

### 2.8 CircSATB1 is Involved in the Activation of the mTOR Signaling Pathway via FKBP8

**Figure 9:**
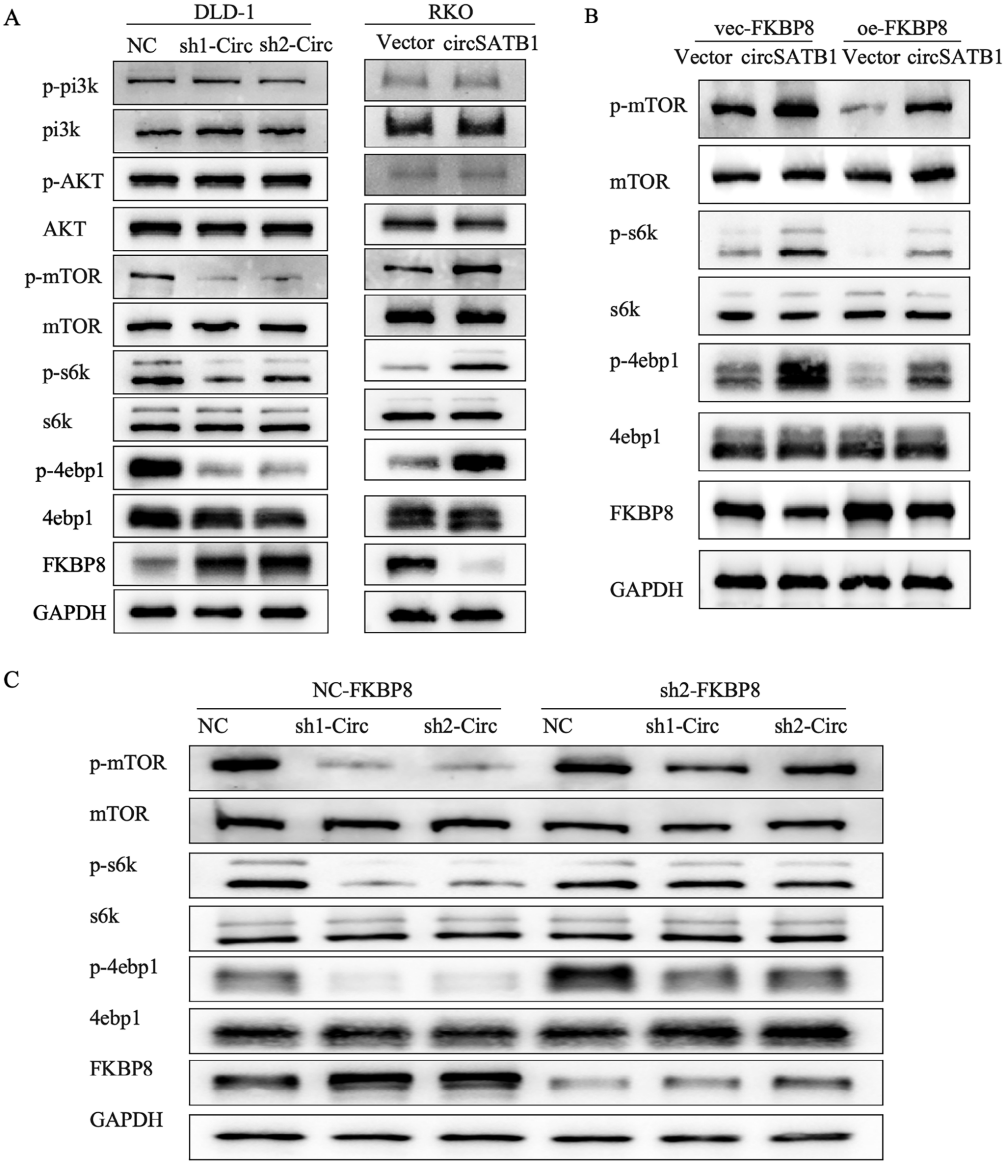
CircSATB1 is involved in the activation of mTOR signaling pathway via FKBP8. A) The effects of NC, sh1‐Circ, sh2‐Circ on mTOR signaling in DLD‐1 cells and effects of Vector, circSATB1 on mTOR signaling in RKO cells detected by WB. B) The reversal effects of FKBP8 on mTOR signaling in RKO cells detected by WB. C) The reversal effects of FKBP8 on mTOR signaling in DLD‐1 cells detected by WB.

Bai et al. identified that FKBP8 is an endogenous inhibitor of mTOR and this inhibition effect was counteracted by Rheb‐GTP in response to growth factor stimulation and nutrient availability.^[^ ^19^, ^20^ ^]^ Hsu et al. disclosed that SPP could promote lung cancer and breast cancer progression by facilitating the degradation of FKBP8 to enhance mTOR signaling.^[^ ^21^ ^]^ To confirm whether circSATB1 could regulate the metastasis of CRC cells via activating mTOR signaling through the proteasomal degradation of FKBP8, WB was performed to detect the activity of mTOR signals. The phosphorylation levels of mTOR, S6K, and 4E‐BP1 were lower in sh1‐circ and sh2‐circ DLD1 cells than NC group. However, there were no significant differences in the levels of p‐PI3K and p‐AKT among the three groups. In the overexpressed circSATB1 RKO cells, only the phosphorylation levels of mTOR, S6K, and 4E‐BP1 were increased significantly (**Figure** **9**A). To evaluate whether FKBP8 mediates the regulation of the mTOR pathway by circSATB1, we conducted rescue experiments.

The WB results demonstrated that FKBP8 could reverse the alterations in the phosphorylation levels of mTOR, S6K, and 4E‐BP1 that were induced by circSATB1 in CRC cells. (Figure 9B,C). These data indicated that circSATB1 was involved in the activation of the mTOR pathway via facilitating FKBP8 proteasomal degradation in CRC cells.

### 2.9 Exosomal circSATB1 in the Plasma is Correlated with CRLM

**Figure 10:**
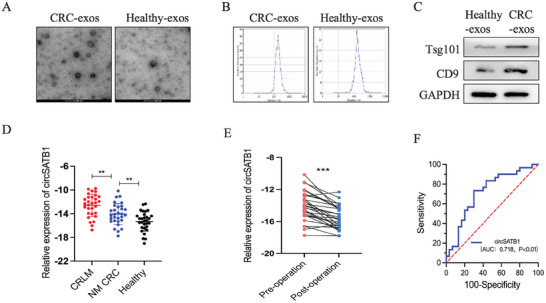
Exosomal circSATB1 in the plasma is correlated with CRLM. A, B) The shape and size of exosomes derived from the plasma of CRC patients and healthy controls were measured by transmission electron microscope (TEM) and Nan tracer. C) The exosome marker of CD9 and Tsg101 was detected by WB. D) The exosomal circSATB1 level in the plasma of CRLM, NM‐CRC patients, and healthy controls were detected by qRT‐PCR(‐ΔCT). E) The exosomal circSATB1 level in the plasma of NM‐CRC patients before and after the radical operation was detected by qRT‐PCR(‐ΔCT). F) AUC value was calculated as a measure of the sensitivity and specificity of the exosomal circSATB1 in CRLM patients. Data are presented as mean ± SD in at least three independent experiments. *P* < 0.05 is considered statistically significant, ^**^ *P* < 0.01, ^***^ *P* < 0.001. One‐way ANOVA test (C) and Student's t‐test (D) were used to determine statistical significance.ROC curve was used to assess the sensitivity and specificity of the exosomal circSATB1 in CRLM.

Numerous studies have revealed that circRNAs can be transferred via exosomes to regulate kinds of cancers.^[^ ^22^ ^]^ To detect whether exosomal circSATB1 participates in the CRLM progression, we extracted exosomes from the plasma of 60 CRC patients(30 CRLM patients,30 NM patients) and 30 healthy controls in cohort 4. Conventionally, the shape and size of the exosomes were measured by transmission electron microscope (TEM) and Nan tracer (**Figure** **10**A,B). The exosome markers were detected by WB (Figure 10C). The qRT‐PCR results showed that the level of exosomal circSATB1 in the plasma of CRLM patients was higher than the NM‐CRC patients (Figure 10D). Notably, the level of exosomal circSATB1 in plasma was reduced significantly after the radical operation for the primary tumor in NM‐CRC patients (R0 resection, n = 30) (Figure 10E). These data suggested that exosomal circSATB1 was secreted from the CRC primary tumor into the plasma and was significantly correlated with the progression of liver metastasis. To evaluate the sensitivity and specificity of exosomal circSATB1 for the diagnosis of CRLM, we performed a receiver operating characteristic (ROC) curve analysis for the 60 CRC patients. The results showed that the areas under the ROC curve (AUC) were 0.718(95% CI, 0.5857 to 0.8499, *P* < 0.01), with 73.33% sensitivity and 70.00% specificity (Figure 10F). These data suggested that exosomal circSATB1 has a certain diagnostic value in CRLM. Thus, exosomal circSATB1 secreted from the CRC primary tumor might play a catalytic role in the progression of CRLM.

## 3 Discussion

According to the data on global cancer in 2020 released by the International Agency for Research on Cancer, there are ≈19.3 million new cancer cases worldwide and ≈10 million cancer deaths. The incidence rate of CRC is 10%, and the mortality rate is 9.4%. It ranks third and second respectively among all malignant tumors, seriously endangering human health.^[^ ^23^ ^]^ Colorectal cancer liver metastasis (CRLM) is the most common type of distant metastasis in CRC and ≈30%–50% of patients will develop liver metastasis during the disease process.^[^ ^3^ ^]^ Among them, the incidence of synchronous liver metastasis is ≈15%–25%, and the incidence of metachronous liver metastasis is ≈18%–25%.^[^ ^3^ ^]^ However, the treatment effects for patients with CRLM are far from satisfactory. As many as 80%–90% of liver metastases cannot be radically resected and the median survival time of CRLM patients without any treatment is only 6.9 months. Even with treatments such as chemotherapy and surgery, the 5‐year survival rate is only ≈10%.^[^ ^3^, ^24^ ^]^ Therefore, it is necessary to deeply understand the molecular mechanism of CRLM and explore new therapeutic targets to improve the prognosis of CRLM.

Circular RNA is a type of RNA with a covalently closed circular structure formed by reverse splicing of precursor messenger RNA (pre‐mRNA). Due to its special structure, circRNA is not easily interfered with by RNAases and is more stable than the linear RNA.^[^ ^25^, ^26^, ^27^, ^28^, ^29^ ^]^ Recent studies have uncovered the significant roles of numerous circRNAs in diverse biological processes.^[^ ^6^, ^30^ ^]^ Especially in cancers, circRNAs have been confirmed to be involved in regulating tumorigenesis and malignant progression.^[^ ^31^, ^32^ ^]^

SATB1 (Special AT‐rich sequence binding protein 1) is a nuclear matrix‐binding protein that can selectively bind to AT‐rich DNA sequences. It regulates chromatin structure and gene expression through high‐order chromatin organization, thereby affecting cell proliferation, apoptosis, invasion, and other functions.^[^ ^33^ ^]^ SATB1 has been confirmed to be closely related to the progression of a large number of malignant tumors and also exerts an extremely crucial role in the progression of CRC.^[^ ^34^ ^]^ In addition to regulating tumor progression through their transcriptional regulation, some oncogenes can splice out circRNAs to exert a function through sequence amplification, deletion, or translocation.^[^ ^35^, ^36^, ^37^, ^38^ ^]^ The regulatory role of SATB1 in CRC is relatively clear. In addition to playing a regulatory role through its encoded protein, it is worth exploring whether SATB1 can affect the progression of CRC by splicing circRNAs. In the present study, we identified circSATB1 (hsa_circ_0064557) as a crucial promoter in CRLM. For the first time, we demonstrated that circSATB1 is overexpressed in CRC tissues and cell lines. Among the clinicopathological characters, the elevated expression of circSATB1 is statistically associated with metastatic factors such as TNM stage, vascular and nerve invasion, and the CEA level.

Notably, clinical analysis showed that circSATB1 overexpression is significantly correlated with the progression of CRLM and shorter survival time.

CircRNAs can exert their functions through various mechanisms in cells. In the nucleus, circRNAs can bind to specific DNA sequences, thereby achieving regulation of the genome.^[^ ^39^, ^40^ ^]^ In the cytoplasm, some circRNAs can act as competing endogenous RNAs (ceRNAs). By binding to miRNAs, they prevent miRNAs from binding to target mRNAs and inhibit the expression of target mRNAs.^[^ ^41^ ^]^ Certain other circRNAs can interact with various proteins, altering the structure or function of these binding proteins, recruiting them to specific sites, or forming circRNA‐protein complexes to fulfill specific roles.^[^ ^42^, ^43^ ^]^ A few circRNAs possess internal ribosome entry sites (IRES) that enable them to undergo cap‐independent translation, resulting in the production of polypeptides or proteins.^[^ ^44^ ^]^ In this study, we initially confirmed the promoting effects of circSATB1 on the metastatic ability of CRC in vivo and in vitro. To investigate the molecular mechanisms underlying the role of circSATB1 in CRC cells, we conducted RNA pulldown, MS, and WB experiments, which revealed that circSATB1 can specifically bind to the FKBP8 protein. To determine the specific binding region of FKBP8 and circSATB1, we constructed truncated plasmids of the FKBP8 and confirmed that domain 2 (120‐220aa) of FKBP8 is the key region interacting with circSATB1.

FK506 binding proteins (FKBPs) are a family of proteins that could be binded with FK506, mediating immunosuppressive procession in eukaryotes. Prolyl cis/trans isomerase (PPIase) domains were the founding domains of FKBPs and were essential for their function.^[^ ^45^, ^46^ ^]^ In addition to PPI, most of the FKBPs possess other functional domains like the tetratricopeptide (TPR) domain, Ca2+/Calmodulin (CaM) binding domain, and so on. FKBPs could regulate a variety of physiological processes including protein folding, cell cycle, apoptosis, autophagy, etc.^[^ ^47^ ^]^ FKBP8 is a multifunctional protein among FKBPs and plays pivotal roles in physiological and disease processes. It has been shown that FKBP8 could exert pro‐ or anti‐apoptotic effects by interacting with the apoptosis regulatory proteins like Bcl2 and Bcl‐xL in different manners.^[^ ^48^, ^49^, ^50^ ^]^ Anchored in the outer mitochondrial membrane, FKBP8 could recruit lipidated LC3A to damaged mitochondria in an LIR‐dependent manner and induce Parkin‐independent mitophagy.^[^ ^51^ ^]^ Additionally, FKBP8 could also form a complex with Hsp90 and NS5A to regulate HCV RNA replication.^[^ ^52^ ^]^ In HCT 116 cells, FKBP8 has been shown to inhibit proliferation by binding to PRL‐3 and promoting its downregulation via the proteasome pathway.^[^ ^53^ ^]^ Additionally, previous studies have shown that FKBP8 can reduce the metastatic progression of 4T1 tumors and B16‐F10 tumors in syngeneic mice.^[^ ^54^ ^]^ In this study, we focused primarily on the role of FKBP8 in mTOR signaling and tumor metastasis, as reported in previous studies.^[^ ^19^, ^20^ ^]^ For the molecular mechanisms, the FKBP8 protein was discovered as one specific binding partner for circSATB1.The protein level of FKBP8, rather than its mRNA level, was altered dramatically by circSATB1. In vitro and vivo, the metastasis‐modulating effects of circSATB1 on CRC cells, either inhibitory or promotional, could be reversed by FKBP8. These data preliminarily suggested that circSATB1 physically combined with FKBP8 protein to facilitate its degradation in a post‐transcriptional manner, thereby promoting the metastasis of CRC cells.

To delve into the precise molecular mechanisms by which circSATB1 regulates the FKBP8 protein at the post‐transcriptional level, we initially subjected CRC cells to treatment with specific inhibitors targeting the ubiquitin‐proteasome system (UPS) and the autophagy‐lysosomal system. Subsequently, by measuring the half‐life of FKBP8, we determined that circSATB1 primarily modulates the level of FKBP8 protein through the ubiquitin‐proteasome system (UPS). Ubiquitylation is a crucial protein posttranslational modification (PTM) process, wherein the ubiquitin (ub) molecule comprising 76 amino acids is conjugated to substrate proteins. This ub molecule features seven distinct lysine residues (K6, K11, K27, K29, K33, K48, and K63), enabling various linking patterns with the substrate protein, thereby executing diverse biological functions.^[^ ^55^, ^56^ ^]^ As the k48‐linked ubiquitin chain is one important signal that mediates 26S proteasomal proteolysis, we got that circSATB1 mainly regulated FKBP8 protein degradation via k48‐linked ubiquitylation.^[^ ^57^, ^58^ ^]^

Ubiquitylation is commonly mediated by 3 enzymes: ubiquitin‐activating enzyme (E1), ubiquitin‐conjugating enzyme (E2), and ubiquitin ligase (E3).^[^ ^59^ ^]^ Among the ubiquitin enzymes, E3s play a crucial role in attaching ub to target proteins and regulating various tumors by modulating the stability of key proteins.^[^ ^60^ ^]^ Based on a comprehensive analysis of the previous experimental results, we found that the E3 ubiquitin ligase RNF25 may participate in the ubiquitination of FKBP8 by binding to circSATB1 and FKBP8, thereby regulating the biological functions of CRC cells.

RNF25 is an E3 ubiquitin‐protein ligase with a RING finger motif involved in the ubiquitination of eEF1A and a discrete set of ribosomal proteins.^[^ ^61^ ^]^ RNF25 is known to promote liver cancer metastasis by mediating the degradation of E‐cadherin protein under oxidative stress conditions.^[^ ^62^ ^]^ In CRC, RNF25 has been shown to cooperate with CARM1 to suppress ferroptosis by binding to ACSL4 and promoting its ubiquitylation and degradation.^[^ ^63^ ^]^ However, the specific role of RNF25 in CRLM has not been previously investigated.

Combining with RNA binding proteins (RBP) is a crucial way for circRNAs to perform their biological functions. There are mainly the following modes: binding to two proteins simultaneously to enhance the interaction between the two proteins; binding to two proteins that have already interacted to relieve the interaction between the two proteins; binding to one protein to enhance or relieve the interaction between this protein and another protein.^[^ ^64^ ^]^ In this study, we discovered that circSATB1 functions as a molecular scaffold, facilitating the formation of a ternary complex with RNF25 and FKBP8. By enhancing the interaction between RNF25 and FKBP8, circSATB1 promotes the degradation of FKBP8 protein via the UPS pathway, specifically mediated by the E3 ligase RNF25. This process leads to a reduction in FKBP8 protein levels at the post‐transcriptional level, which in turn alleviates its inhibitory effects on the mTOR pathway and promotes the progression of CRLM.

It has been demonstrated that circRNAs can be loaded and transported by exosomes to regulate various physiological and disease processes.^[^ ^65^, ^66^, ^67^ ^]^ Owing to the advantage of excellent non‐invasive extraction and detection, serum exosomal circRNA has the potential to act as a biomarker for early diagnosis, prognosis analysis, and the like.^[^ ^68^ ^]^ For example, Exosomal circCOL1A1 derived from CRC cells promotes the migration ability of tumor cells by recruiting EIF4A3 and activating the Smad2/3 signal.^[^ ^69^ ^]^ Exosomal circTUBGCP4 can adsorb miR‐146b‐3p and upregulate PDK2 to activate the AKT signaling pathway. It enhances the migration ability of vascular endothelial cells and angiogenesis by inducing the formation of filopodia and endothelial cells, thereby facilitating CRC metastasis.^[^ ^70^ ^]^ In this study, we observed that exosomal circSATB1 was upregulated in the plasma of CRLM patients than the NM‐CRC patients. Notably, the level of exosomal circSATB1 in plasma was reduced significantly after the radical operation for the primary tumor in NM‐CRC patients. The ROC curve analysis for the 60 CRC patients showed that AUC was 0.718, with 73.33% sensitivity and 70.00% specificity. Thus, exosomal circSATB1 secreted from the primary tumor of CRC is likely to play a catalytic role in the progression of CRLM and has a certain diagnostic value for CRLM.

In summary, this study demonstrates that circSATB1 promotes the progression of CRLM by facilitating the ubiquitylation and degradation of FKBP8, releasing its inhibitory effects on mTOR signaling. In this process, RNF25 functions as an E3 ubiquitin ligase, mediating K48‐linked ubiquitylation of FKBP8, which is further augmented by circSATB1 as a scaffold for RNF25‐FKBP8 complexes. Notably, circSATB1 can be secreted from the CRC primary tumor by exosomes into plasma and is closely related to CRLM. These findings provide novel insights and potential therapeutic targets for CRLM. However, there are still certain limitations in this study. The study did not explore clinical drugs or interfering molecules targeting the ternary complex of RNF25‐circSATB1‐FKBP8, and further work in this regard needs to be carried out. This study focused on the expression level and diagnostic value of exosomal circSATB1 in the plasma for patients with CRLM. In future research endeavors, we will persist in investigating the mechanisms of exosomal circSATB1 in CRLM.

## 4 Experimental Section

### Samples

Four independent cohorts of CRC patients treated at the Department of Colorectal Surgery of the First Affiliated Hospital of Nanjing Medical University were included in this study. All the information on the patients in the four cohorts is shown in Table S5 (Supporting Information). Cohort 1, containing randomized 24 CRC patients (12 LM patients, 12 NM patients), was applied to screening for differentially expressed circRNAs. Cohort 2, including randomized 120 CRC patients with or without liver metastasis, was applied to analyze circSATB1 expression level with the clinical characters. Cohort 3, including randomized 56 CRC patients with or without liver metastasis, was applied to analyze protein levels of FKBP8 and RNF25 with the clinical characters. Cohort 4, intaking randomized 60 CRC patients (30 LM patients, 30 NM patients) and 30 healthy people, was applied to detect the relationship of exosomal circSATB1 level with CRLM. Tumor and matched normal tissues were obtained from the patients in Cohort 1–3. All the tumors and matched normal tissues were stored in liquid nitrogen immediately after the surgical resection. Venous blood was obtained from the patients before any kind of therapy and healthy controls in Cohort 4. This research was approved by the Ethics Committee of The First Affiliated Hospital of Nanjing Medical University with approval number 2023‐SR‐213. The informed consent was obtained from all participants or next of kin, as appropriate.

### Cell Culture

SW480, LoVo, HT‐29, DLD‐1, RKO, HCT116 cell lines, normal human colon epithelial cell line(FHC), and HEK‐293T cell lines were purchased from the National Collection of Authenticated Cell Cultures and American Type Culture Collection(ATCC). All cell lines were cultured in a humidified atmosphere containing 5% CO2 at 37 °C with the recommended medium.

### Cell Transfection

The lentivirus containing targeting gene sequences, short hairpin RNAs (shRNAs) (Table S6, Supporting Information), and empty vectors were purchased from Obio (Shanghai, China). For stable gene interference effects, stable and reliably viable cell lines were constructed through lentivirus infection. Lentivirus infection was mediated with a serum‐free medium containing 5µg mL^−1^ of polybrene(Millipore, USA, TR‐1003‐G) for 16 h. Next, cells were cultured with medium containing serum and screened with 1–6µg mL^−1^ Puromycin (InvivoGen, ant‐pr‐1). The surviving cells were cultured and used for interference efficiency through qRT‐PCR and WB and for other experiments. The plasmids for wild‐type ubiquitin and the mutants were synthesized by Genomeditech(Shanghai, China). The truncated plasmids of FKBP8(1‐220aa, 1‐119aa+221‐412aa, 120‐412aa), wild‐type, and the mutants of FKBP8 were obtained from Obio(Shanghai, China). Cells were cultured in 6‐well plates and transfected with 5 µg plasmids accompanied with 5 µL. Lipofectamin3000(Invitrogen, L3000001) in 250 µL serum‐free medium for 6h. Next, cells were cultured with medium containing serum for 24–78 h and then used for further experiments.

### Fluorescence In Situ Hybridization (FISH) Assay

The FISH Probe Mix of circSATB1 was designed by Tsingke Biotech (Beijing, China) (Table S6, Supporting Information). The FISH Probe Mix was used to detect the cell localization of circRNAs combined with a Fluorescent In Situ Hybridisation Kit(RiboBio, China, R11060). The cells in confocal dishes were immobilized by 4% formaldehyde for 10 min at RT and then were incubated by enhanced immunostaining permeabilization buffer(Beyotime, China) for 10 min. The cells were blocked by pre‐hybridization at 37 °C and then incubated with 100µL hybridization containing 2.5 µL 20 µm FISH Probe Mix overnight at 37 °C in the dark. After being washed by 4×,2×,1×SSC buffer and PBS, the cells were dyed using DAPI Staining Solution(Beyotime, China, C1005) for 10 min at RT. All the dishes were photographed by the Stellaris STED Laser confocal microscopy(LEICA, Germany).

### Immunofluorescence (IF)

The treated CRC cells grown in confocal dishes were fixed with 4% paraformaldehyde for 10 min and permeabilized with Enhanced Immunostaining Permeabilization Solution(Beyotime, China, P0097) or Immunostaining Permeabilization Solution with Saponin(Beyotime, China, P0095) for 10 min. Next, the cells were dealt with Immunol Staining Blocking Buffer(Beyotime, China, P0102) at RT for 60 min. The cells were then incubated with specific primary antibodies for FKBP8 and RNF25 at 4 °C overnight and incubated with fluorescent secondary antibodies at RT for 1 h. All the dishes were photographed by the Stellaris STED Laser confocal microscopy(LEICA, Germany).

### RNA Pull‐Down Assay, Mass Spectrometry(MS), and Silver‐Staining Assay

The probes targeting the back‐splicing site of circSATB1 were designed and synthesized by RiboBio (Guangzhou, China). The pull‐down probe sequences are listed in Table S6 (Supporting Information). The pull‐down assays were performed using the biotin‐labeled probes and the Pierce Magnetic RNA‐Protein Pull‐Down Kit (Thermo Fisher Scientific, USA, 20164) according to the manufacturer's instructions. The extracted binding proteins were identified by mass spectrometry (BGITech Company, Shen Zhen, China). The precipitates were also analyzed through a silver‐staining assay using Fast Silver Stain Kit (Beyotime, China, P0017S) according to the protocol.

### RNA‐Binding Protein Immunoprecipitation (RIP) Assay

All steps and instruments involved in the procedure were meticulously ensured to be free of both DNase and RNase contamination. The RIP assay was conducted using the Magna RIP Kit sourced from Millipore (USA, 17–700). Here's a concise overview of the key steps: 1) Lysate Preparation: Cells were incubated on ice with RIP Lysis Buffer, supplemented with 0.5 µL of protease inhibitor cocktail and 0.25 µL of RNase inhibitor per 100 µL of buffer. The 10 µL supernatant obtained from this lysate served as the “Input” sample. 2) Preparation of Magnetic Beads for Immunoprecipitation: 5 µg of a specific antibody was incubated with the prepared magnetic beads for 30 min at RT with rotation. Following two washes with RIP Wash Buffer, the magnetic beads were resuspended in 0.5 mL of RIP Wash Buffer. 3) Immunoprecipitation: Each 1.0 mL aliquot of the immunoprecipitation reaction mixture contained the beads‐antibody complex, accompanied by 900 µL of RIP Immuno‐precipitation Buffer and 100 µL of RIP lysate supernatant. The mixture was rotated at 4 °C overnight. Subsequently, the beads were washed six times with 0.5 mL of RIP Wash Buffer. Proteins and RNAs could then be purified and isolated from the washed beads. 4) Detection: The immunoprecipitated RNA or proteins were detected using qRT‐PCR or WB, respectively.

### Immunoprecipitation (IP) and Co‐Immunoprecipitation (Co‐IP) Assay

IP and Co‐IP assays were performed with Pierce Co‐Immunoprecipitation Kit(Thermo Fisher Scientific, USA, 26149). The assays were performed according to the protocols to verify the binding of proteins with each other. The IP/Co‐IP products were detected by WB.

### Ubiquitination Assay

Cells transfected with established plasmids(wild‐type ubiquitin and the mutants(K6, K11, K27, K29, K33, K48, K63), Flag‐FKBP8, sh‐Circ, CircSATB1, His‐RNF25, sh‐RNF25, truncated plasmids of FKBP8(1‐220aa, 1‐119aa+221–412aa, 120‐412aa), plasmids of wild‐type FKBP8 or mutants(K249R,K271R, K273R, K284R, K307R, K314R, K334R, K340R, K348R, K366R, K377R) stably were pretreated with proteasome inhibitor MG‐132 (Beyotime, China, S1748) at a concentration of 20 µm for 8 h before harvesting. IP and IB were performed with the specific antibody to detect the ectogenic or endogenic ubiquitination levels.

### Animal Models

All the animal experiments in this study were approval by the Institutional Animal Care and Use Committee (IACUC) of Nanjing Medical University with approval number IACUC‐2211039. The 6‐week‐old BALB/c nude mice were obtained from Gempharmatech and fed in the Animal Core Facility of Nanjing Medical University. To detect the CRC cell's metastasis capacity in vivo, luciferase‐labeled transfected cells (1×10^6^/100 µL PBS) were injected into the distal tip of the spleen under general anesthesia to build liver metastasis models. Four weeks later, the metastatic foci in vivo were visualized by an IVIS Spectrum Imaging System (PerkinElmer, USA) after injecting D‐luciferin (150 mg kg^−1^) (Goldbio, USA, LUCK‐1G) intraperitoneally in mice. The liver specimens were immobilized by formalin and the pathological property was confirmed by HE staining. Overall survival was recorded at 10 weeks.

### Statistical Analysis

Statistical analysis was performed by R 4.1.1, GraphPad Prism 8.0, ImageJ 1.52, and spss 25.0 software. Paired or unpaired t‐tests, ANOVA tests, and Chi‐square tests were used to determine statistical significance in this study. Kaplan–Meier curves were used to show the OS and PFS and log‐rank tests were used to determine statistical significance. Each experiment was repeated at least three times independently. Data were shown as means ± standard deviation (SD). Differences were considered statistically significant when *P* < 0.05. ^*^ *P* < 0.05, ^**^ *P* < 0.01 and ^***^ *P* < 0.001. Differences were considered not significant (n.s.) when *P* > 0.05.

## Conflict of Interest

The authors declare no conflict of interest.

## Supporting information



Supporting Information

Supplemental Table 1

Supplemental Table 2

Supplemental Table 3

Supplemental Table 4

Supplemental Table 5

Supplemental Table 6

## Data Availability

The data that support the findings of this study are available from the corresponding author upon reasonable request.
