# Supplementary material for: CircSATB1 Promotes Colorectal Cancer Liver Metastasis through Facilitating FKBP8 Degradation via RNF25‐Mediated Ubiquitination
Source: Adv Sci (Weinh). 2025 Feb 8;12(13):2406962. doi: 10.1002/advs.202406962 (PMC11967755; doi:10.1002/advs.202406962)
Supplement: Supplementary file 1 — Supporting Information [file ADVS-12-2406962-s002.docx]

**Supporting Information**

**CircSATB1 promotes colorectal cancer liver metastasis through facilitating FKBP8 degradation via RNF25-mediated ubiquitination**

Chuan Zhang,Chuanxin Tian,Renzhong Zhu,Chen Chen,Chi Jin,Xiaowei Wang,Lejia Sun,Wen Peng,Dongjian Ji,Yue Zhang,and Yueming Sun*

Chuan Zhang,Chuanxin Tian,Renzhong Zhu,Chen Chen contributed equally to this work.

*Corresponding author:Yueming Sun,sunyueming@njmu.edu.cn

**This supporting information includes:**

Supporting Methods.

Figure S1 to S4.

**Supporting Tables (see Excel files)：**

Table S1.Associations between the expression of hsa_circ_0064557 and clinical factors of CRC patients in cohort 2.

Table S2.12 proteins within 35 kDa and 70kDa in DLD-1 and RKO cells.

Table S3.Associations between the protein level of FKBP8 and clinical factors of CRC patients in cohort 3.

Table S4.Associations between the protein level of RNF25 and clinical factors of CRC patients in cohort 3.

Table S5.The information of the patients in four cohorts.

Table S6.Sequences in this study.

**Supporting Methods**

RNA extraction and quantitative real-time polymerase reaction(qRT-PCR):Total RNA of tissues and cells was isolated using TRIzol reagent (Thermo Fisher Scientific, USA,15596026).The concentration and purity of the total RNA was measured by NanoDrop 2000 Spectrophotometer(Thermo Fisher Scientific, USA).cDNA was reversely transcribed utilizing PrimeScript RT Master Mix (Perfect Real Time) (Takara,RR036A) or HiScript III RT SuperMix for qPCR(Vazyme, China,R323).qRT-PCR was performed with AceQ qPCR SYBR Green Master Mix (High ROX Premixed)(Vazyme, China,Q141) on an Applied Biosystems StepOnePlus RT-PCR System.GAPDH was used as the normalizing control.The products were separated by 2% agarose gels. Primers were designed and synthesized by Tsingke Biotech(Beijing, China) and RiboBio (Guangzhou, China).The primers are listed in Table S6(Supporting Information).

RNase R treatment:Total RNA (2 μg) was incubated with 6U RNase R(Beyotime, China,R7092 ) at 37 °C for 30 min and inactivated at 70°C for 10 min.After purification and collection,the resulting RNA was reversely transcribed and analysed by qRT-PCR.

## Reagent treatment:The cells were treated with cycloheximide (CHX, 100 μg/mL)(MCE,USA,HY-12320) for the indicated time points before harvesting. The cells were treated with Chloroquine(Aladdin,C193834),3-Methyladenine (3-MA) (Aladdin,M129496) or MG-132(Beyotime,China,S1748) with different concentration showed in the figures.The protein products obtained from the treated cells were detected by WB.

Wound healing assay and Transwell assay:Cells were seeded into six-well plates(1×10^6^ cells/well) and equidistant wound scratches were generated by a 200ul pipette tip after incubation for 24 h.Before and after culturing cells in medium without Fetal Bovine Serum (FBS)(GIBCO,10099141C)for 48h,the width of the scratch were photographed.The data was performed by ImageJ.Transwell chambers (Corning, USA) were used to perform the transwell assay to assess the invasive abilities of the CRC cells.3 × 10^4^ cells in 200 μl fresh medium without FBS were plated into the upper chamber and 700 μL medium with 10% FBS was supplied to the lower section.After incubation for 48 h, the cells on the surface of the chambers were stained with crystal violet for 30 min at RT and then the cells on the supper surface were cleared with a cotton swab. These experiments were performed in triplicate. The number of cells were counted by ImageJ.

Western Blotting(WB)/Immunoblotting(IB):Total proteins of the tissues and cells were extracted by RIPA lysis buffer (Beyotime, China,P0013B) containing Protease inhibitor cocktail(Beyotime, China,P1006).Protein content was measured with a BCA Protein Assay Kit (Beyotime,China,P0010).Proteins were separated by sodium dodecyl sulfate–polyacrylamide gel electrophoresis and then transferred to polyvinylidene difluoride (PVDF) membranes (Millipore).The PVDF membranes were blocked by Protein Free Rapid Blocking Buffer(EpiZyme,China,PS108) for 30min at RT, incubated with specific primary antibodies at 4 °C overnight and incubated with secondary primary antibodies at RT for 2 h.The band densities were quantified by ImageJ.

Antibodies and Reagents:Antibodies for FKBP8(Proteintech,China,11173-1-AP),

FKBP8(Proteintech,China,66690-1-Ig),RNF25(Proteintech,China,24536-1-AP),GAPDH(Proteintech,China,60004-1-Ig),DYKDDDDK tag Polyclonal antibody (Binds to FLAG® tag epitope)(Proteintech,China,20543-1-AP),LYN(Proteintech,China,

18135-1-AP),Tsg101(Proteintech,China,28283-1-AP),CD9(Proteintech,China,20597-1-AP),RNF25(Sigma-Aldrich,USA,HPA036420),AKT(Cell Signaling Technology,

USA,9272),p-AKT(Cell Signaling Technology,USA,9271),mTOR(Cell Signaling Technology,USA,2983),p-mTOR(Cell Signaling Technology,USA,5536),4-EBP1

(Cell Signaling Technology,USA,9644),p-4-EBP1(Cell Signaling Technology,USA,

2855),s6K(Cell Signaling Technology,USA,2708),p-s6K(Cell Signaling Technology,

USA,9234),Argonaute2(Cell Signaling Technology,USA,2897),Ubiquitin(Cell Signaling Technology,USA,3936), E-cadherin(Proteintech,China,20874-1-AP),

Vimentin(Proteintech,China, 60330-1-Ig),HA-Tag(Cell Signaling Technology,

USA,3724),His-Tag(Cell Signaling Technology,USA,12698) were used in this study.Goat Anti-Rabbit IgG (H+L)(Jackson,111-035-003),Goat anti-mouse IgG (H+L)(Jackson,115-035-003),Alexa Fluor 488-labeled Goat Anti-Mouse IgG(H+L)

(Beyotime,China,A0428),Alexa Fluor 555-labeled Donkey Anti-Rabbit IgG(H+L)(Beyotime,China,A0453) were purchased commercially.

Exosome Isolation and identification:Firstly,30ml culture media(1×10^8^ cells)was centrifuged at 300×g for 10 min to remove cells.Secondly,The supernatant was centrifuged at 2000×g for 10 min to remove dead cells and then centrifuged at 10000×g for 30 min to remove cell debris.Lastly,the remaining supernatant was centrifuged at 100000×g for 70 min twice and washed with PBS for one time in the intermediate interval.The pellets were resuspended in 100ul sterile 1×PBS.The exosomes in plasma were extracted by exoRNeasy Serum/Plasma Maxi Kit (Qiagen, Hilden, Germany,77064) according to the protocol.Exosomes were resuspended in 100ul sterile 1×PBS.The shape,size and amount of exosomes were identified by transmission electron microscope (TEM)(FEI,Tecnai G2) and nanoparticle tracking analysis (NTA,Particle Metrix, Germany).

**Supporting Figures**

**
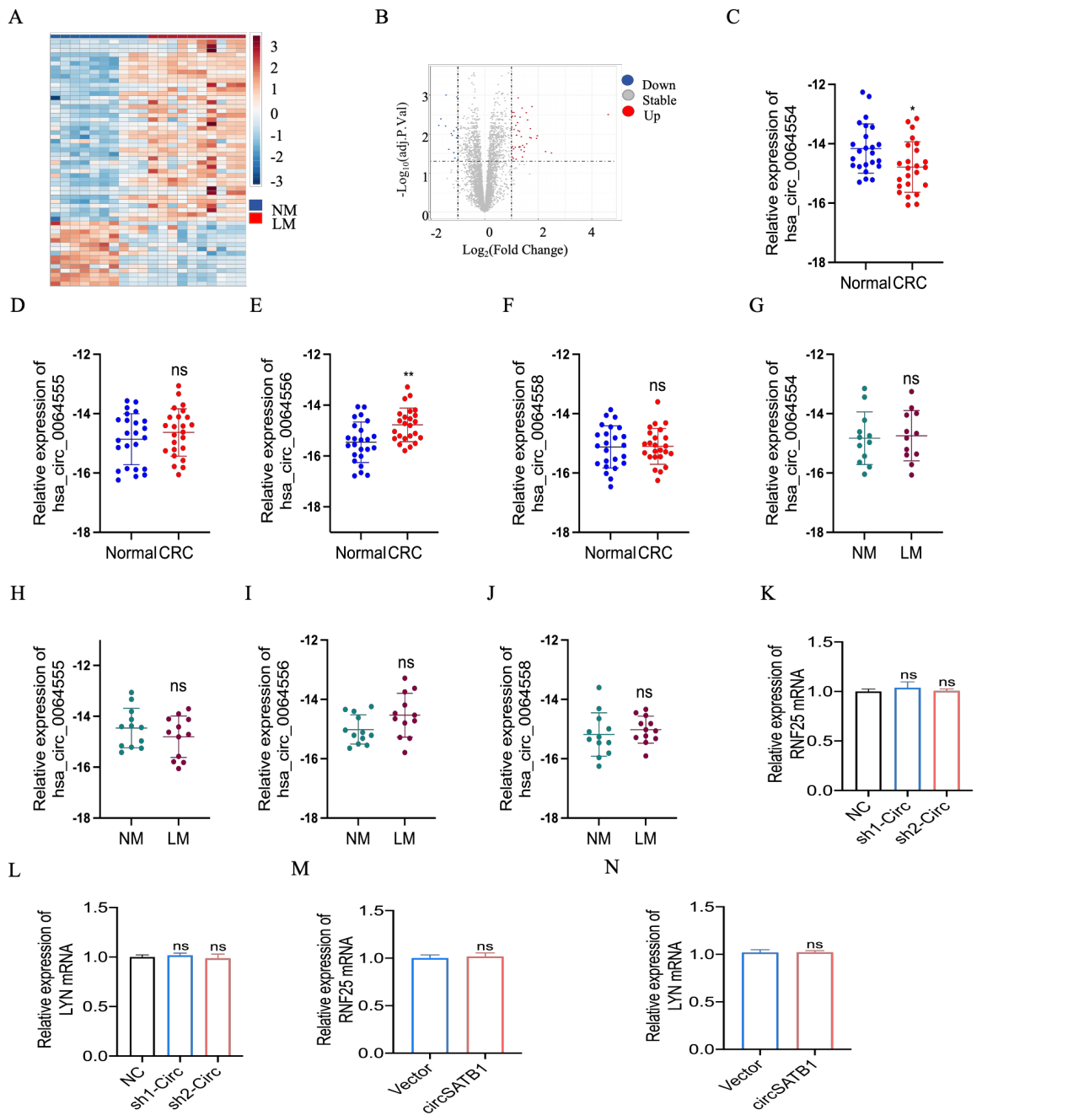
**

**Figure S1.hsa_circ_0064557 is upregulated in CRC tissues with LM and has no effects on RNF25 or LYN mRNA**

(A,B)Heat map and Volcano plots of differentially expressed circRNAs in GSE147597 between the CRC tissues in LM group and NM group(|logFC|>1,adj.P< 0.05).

(C-F)The relative expression level( -ΔCT) of hsa_circ_0064554,hsa_circ_0064555,

hsa_circ_0064556 and hsa_circ_0064558 in adjacent normal tissues and CRC tissues from the 24 patients in cohort 1. n=24 per group.

(G-J)The relative expression level( -ΔCT) of hsa_circ_0064554,hsa_circ_0064555,

hsa_circ_0064556 and hsa_circ_0064558 in CRC tissues from the 24 patients in NM and LM group in cohort 1.n=12 per group.

(K,L)The effects of sh1-Circ and sh2-Circ on the mRNA level of RNF25 and LYN

(2^-ΔΔCT^).

(M,N)The effects of Vector and circSATB1 on the mRNA level of RNF25 and LYN

(2^-ΔΔCT^).

Data are presented as mean ± SD at least three independent experiments.*P < 0.05, **P < 0.01, and ***P < 0.001,P > 0.05, not significant (n.s.).Student’s t test (C-J,M,N) and one-way ANOVA test(K,L) were used to determine statistical significance.


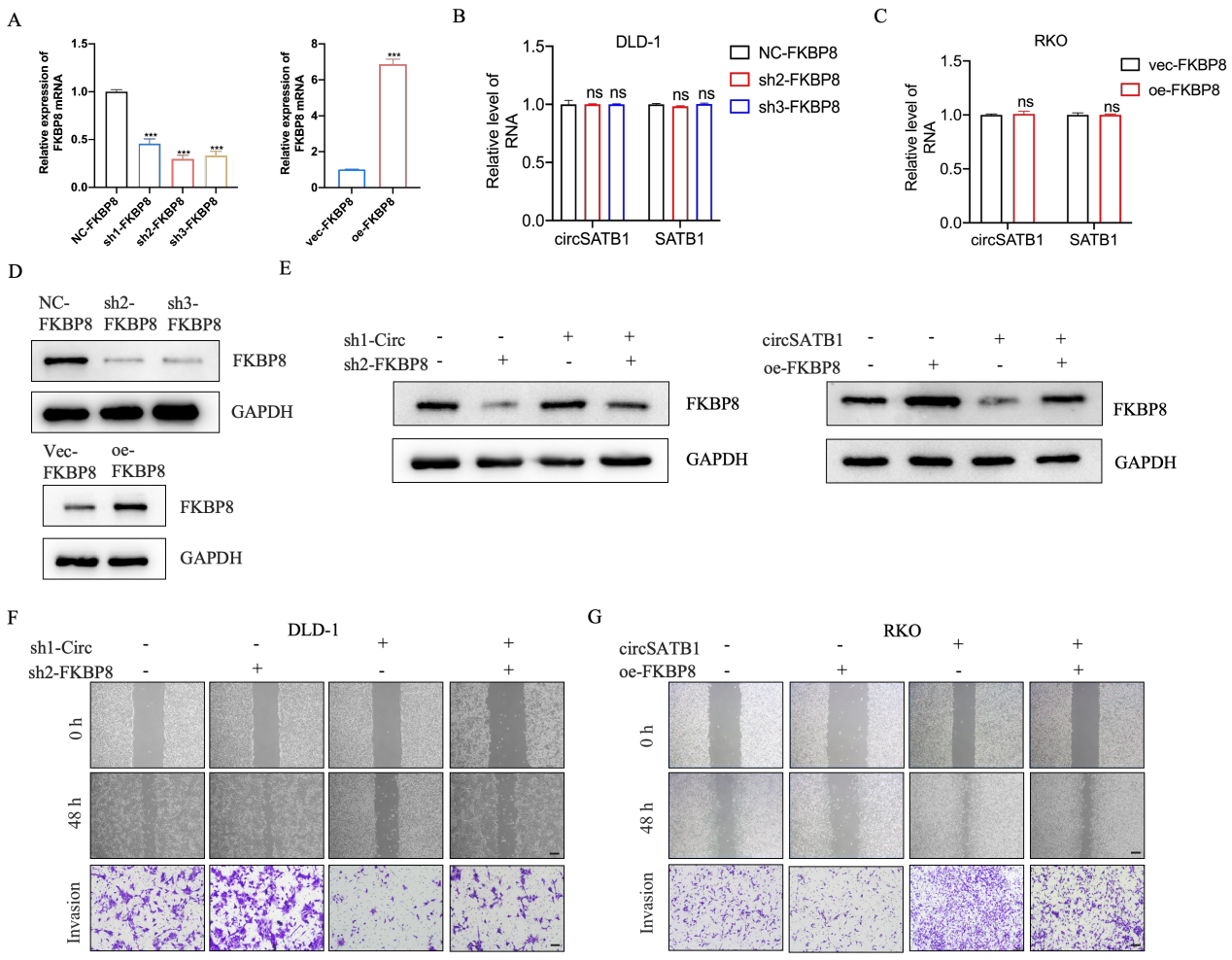


**Figure S2.The verification of the reversal effects of FKBP8**

1. The konckdown and overexpression efficiency of FKBP8 mRNA validated by qRT-PCR(2^-ΔΔCT^).
2. The effects of sh2-FKBP8,sh3-FKBP8 on circSATB1 and linear SATB1 mRNA validated by qRT-PCR(2^-ΔΔCT^).
3. The effects of oe-FKBP8 on circSATB1 and linear SATB1 mRNA validated by qRT-PCR(2^-ΔΔCT^).
4. The konckdown and overexpression efficiency of FKBP8 protein validated by WB.
5. The reversal effects of FKBP8 protein validated by WB.

(F,G)The reversal effects of FKBP8 on the migration and invasion capacity of DLD-1 and RKO cells confirmed by wound healing and transwell assays in vitro(Wound Healing,Scale bar, 200 μm;Transwell,Scale bar, 100μm).

Data are presented as mean ± SD at least three independent experiments.*P < 0.05, **P < 0.01, and ***P < 0.001,P > 0.05, not significant (n.s.).One-way ANOVA test(A left and B) and Student’s t test (A right and C) were used to determine statistical significance.


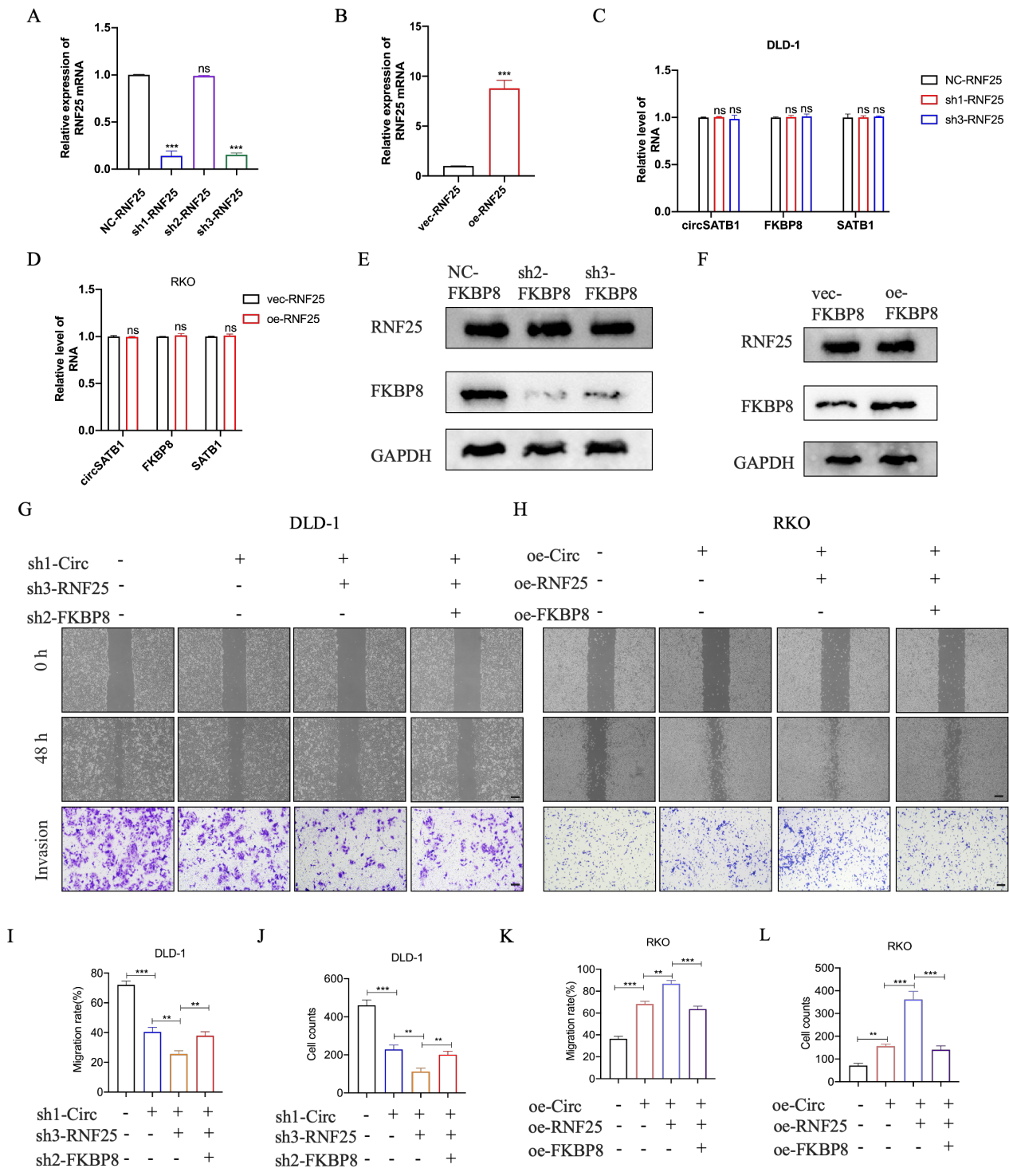
**Figure S3.The verification of the effects of RNF25 on circSATB1,linear SATB1 and FKBP8 mRNA and on the metastastic capabilities of CRC cells**

(A,B)The konckdown and overexpression efficiency of RNF25 mRNA validated by qRT-PCR(2^-ΔΔCT^).

(C)The effects of sh1-RNF25,sh3-RNF25 on circSATB1,linear SATB1 and FKBP8 mRNA validated by qRT-PCR(2^-ΔΔCT^).

(D)The effects of oe-RNF25 on circSATB1,linear SATB1 and FKBP8 mRNA validated by qRT-PCR(2^-ΔΔCT^).

(E,F)The protein level of FKBP8 and RNF25 regulated by FKBP8 knockdown or overexpression.

(G-L)The effects of circSATB1,RNF25 and FKBP8 on migratory and invasive capabilities of DLD-1 and RKO cells validated by wound healing and transwell assays in vitro(Wound Healing,Scale bar, 200 μm;Transwell,Scale bar, 100μm).

Data are presented as mean ± SD at least three independent experiments.*P < 0.05, **P < 0.01, and ***P < 0.001,P > 0.05, not significant (n.s.).One-way ANOVA test

(A,C,I-L) and student’s t test (B,D) were used to determine statistical significance.

**
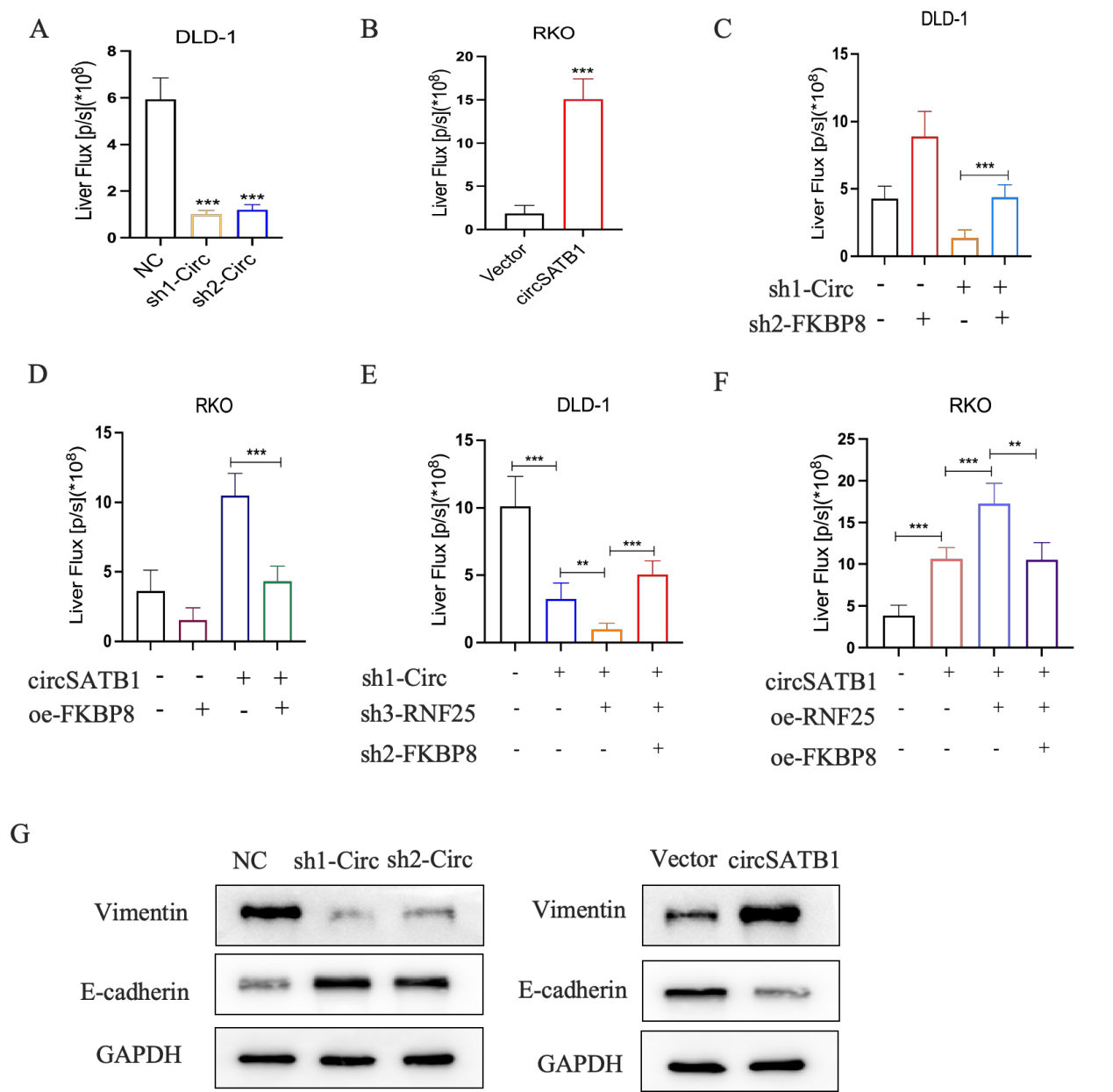
**

**Figure S4. Bioluminescent signals of liver metastasis models in vivo and the effects of circSATB1 on EMT markers**

(A)Bioluminescent signals showed the effects of circSATB1 knockdown on the liver metastasis capabilities of DLD-1 cells in vivo.n=5 per group.

(B)Bioluminescent signals showed the effects of circSATB1 overexpression on the liver metastasis capabilities of RKO cells in vivo.n=5 per group.

(C-D)Bioluminescent signals showed the reversal effects of FKBP8 on the liver metastasis capabilities of DLD-1 and RKO cells in vivo.n=5 per group.

(E-F)Bioluminescent signals showed the synergistic effects of circSATB1,RNF25 and FKBP8 on the liver metastasis capabilities of DLD-1 and RKO cells in vivo.n=5 per group.

(G) The effects of knockdown or overexpression of circSATB1 on the EMT markers.

Data are presented as mean ± SD at least three independent experiments. **P < 0.01, and ***P < 0.001,P > 0.05, not significant (n.s.).One-way ANOVA test(A,C-F) and student’s t test (B) was used to determine statistical significance.
